# ppBAM: ProteinPaint BAM track for read alignment visualization and variant genotyping

**DOI:** 10.1093/bioinformatics/btad300

**Published:** 2023-05-04

**Authors:** Robin Paul, Jian Wang, Colleen Reilly, Edgar Sioson, Jaimin Patel, Gavriel Matt, Aleksandar Acić, Xin Zhou

**Affiliations:** Department of Computational Biology, St. Jude Children’s Research Hospital, Memphis, TN 38105, United States; Department of Computational Biology, St. Jude Children’s Research Hospital, Memphis, TN 38105, United States; Department of Computational Biology, St. Jude Children’s Research Hospital, Memphis, TN 38105, United States; Department of Computational Biology, St. Jude Children’s Research Hospital, Memphis, TN 38105, United States; Department of Computational Biology, St. Jude Children’s Research Hospital, Memphis, TN 38105, United States; Department of Computational Biology, St. Jude Children’s Research Hospital, Memphis, TN 38105, United States; Department of Computational Biology, St. Jude Children’s Research Hospital, Memphis, TN 38105, United States; Department of Computational Biology, St. Jude Children’s Research Hospital, Memphis, TN 38105, United States

## Abstract

**Summary:**

ProteinPaint BAM track (ppBAM) is designed to assist variant review for cancer research and clinical genomics. With performant server-side computing and rendering, ppBAM supports on-the-fly variant genotyping of thousands of reads using Smith–Waterman alignment. To better visualize support for complex variants, reads are realigned against the mutated reference sequence using ClustalO. ppBAM also supports the BAM slicing API of the NCI Genomic Data Commons (GDC) portal, letting researchers conveniently examine genomic details of vast amounts of cancer sequencing data and reinterpret variant calls.

**Availability and implementation:**

BAM track examples, tutorial, and GDC file access links are available at https://proteinpaint.stjude.org/bam/. Source code is available at https://github.com/stjude/proteinpaint.

## 1 Introduction

Mutation detection by next-generation sequencing is becoming mainstream in both cancer research and clinical diagnosis. Common practice includes running variant callers such as GATK (McKenna et al. 2010) for detecting variants. Manual review is essential to ensure variant calling accuracy by visually examining read alignment over mutations, especially for indels and complex mutations with alignment inconsistencies. Current BAM visualization tools such as IGV (Robinson et al. 2017), pileup.js (Vanderkam et al. 2016), BamView (Carver et al. 2013), UCSC Genome Browser BAM track (Fujita et al. 2011), and BamSnap (Kwon et al. 2021) provide limited support for manual review of variant calls (Supplementary Table S1), primarily due to the visualization using only reference genome and lacking support for complex mutations. Here, we present ProteinPaint BAM track (ppBAM), a web-based BAM visualization tool based on the ProteinPaint platform (Zhou et al. 2021), designed for accurate and efficient variant review. ppBAM leverages ProteinPaint’s server-side computing and rendering to be performant for analyzing variant calls from deep sequencing data. It is well-suited to support clinical genomics efforts such as early detection of pathogenic complex mutations and is easily integrated into local or cloud-based clinical genomics workflows to assist manual review. The cancer research community can also benefit from ppBAM through its support of the NCI Genomic Data Commons (GDC) BAM slicing API (Heath et al. 2021).

## 2 Methods

### 2.1 Improved variant genotyping by ppBAM

Typical variant calling results provide only the number of reads supporting the reference and alternative allele, and do not report reads not matching either alleles or uninformative reads with equal similarity to both alleles. As an improvement, ppBAM divides reads to up to four groups against a variant by aligning each read to both reference and alternative alleles using a Rust implementation of Smith–Waterman algorithm (Köster 2016) (Supplementary Fig. S1) that can process thousands of reads on-the-fly (see Supplementary Tutorial). The first two groups are reads supporting the alternative and reference alleles in case of single-allele variants. In case of multiallele variants, multiple groups could be created for each of the alternative alleles (Supplementary Fig. S2). The next group contains reads not supporting either allele which may be due to a wrong base call at the variant region or possibility of presence of a different alternative allele. The last group contains ambiguous reads with equal similarity to both alleles. Reads from each group are displayed separately for visual comparison (Supplementary Fig. S3).

### 2.2 Realignment of reads supporting alternative allele

Reads supporting the complex indels can be challenging to comprehend when viewing their alignments on the reference genome, hindered by alignment inconsistencies between reads which otherwise have the same read sequences in the variant region. To solve this problem, the ppBAM allows the user to realign a group of reads against a mutated reference genome which can clearly display read support for a complex mutation. The realignment is done using multiple-sequence alignment tool Clustal Omega (Sievers and Higgins 2018).

### 2.3 Visualization of cancer genome sequencing results from NCI Genomic Data Commons

ppBAM supports access to human cancer genome sequencing data hosted in NCI GDC. Given a sample or file ID, ppBAM will list BAM files from the sample, as well as somatic mutations of the same sample by querying the GDC API. Users can either select a mutation, or enter a genomic region or custom mutation to view the read alignments from the GDC BAM file. On-the-fly genotyping is performed on a mutation for users to review the read support (Supplementary Fig. S4).

## 3 Use cases

### 3.1 ppBAM genotyping resolves alignment inconsistencies in a *TP53* intragenic deletion case

**Figure 1.**
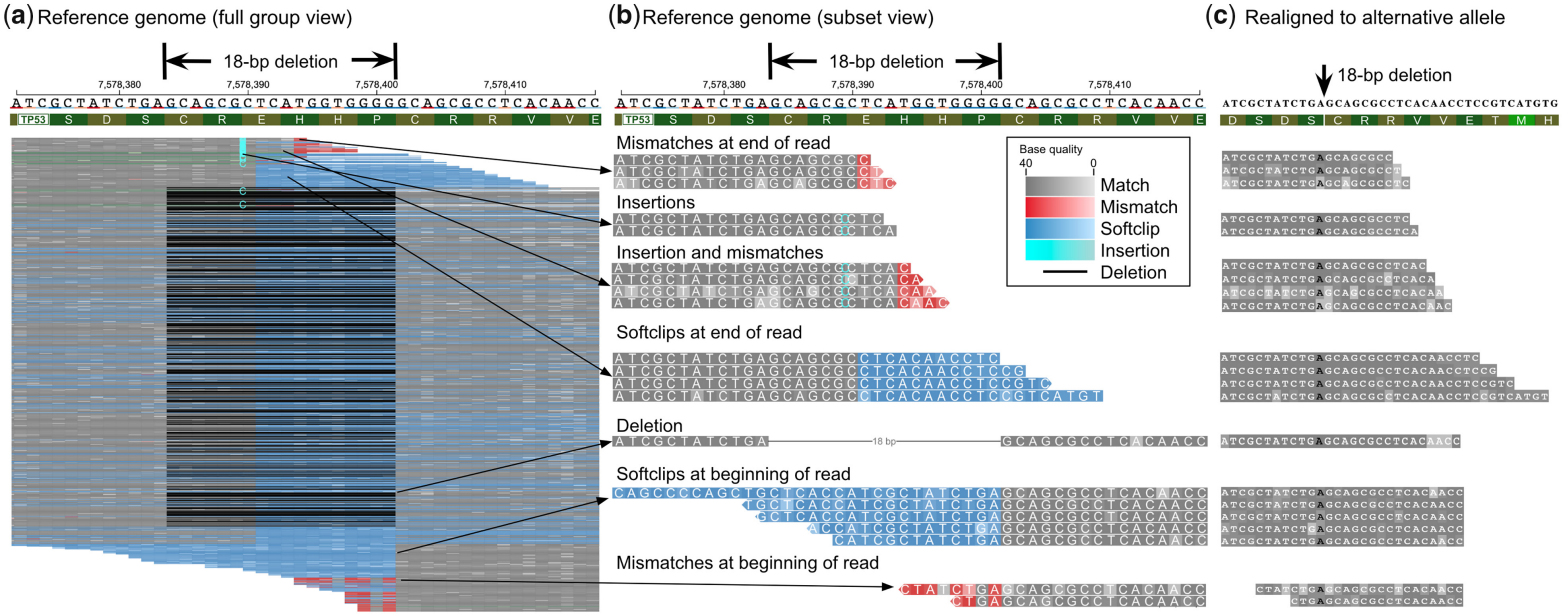
TP53 18-bp deletion example. (a) A complete view of reads supporting the TP53 deletion. (b) Detailed view of selected set of reads from (a) with various alignment inconsistencies. (c) The same set of reads as (b) but realigned to the alternative allele using ClustalO.

At an 18-bp deletion in *TP53*, ppBAM classifies out of a total of 1853 reads, 654 reads as supporting reference allele, 959 as supporting alternative allele, 62 as not supporting either allele, and 178 as ambiguous (Supplementary Fig. S3). Reads classified as supporting alternative allele displays alignment inconsistencies including mismatches, softclips, insertions, or combinations of these (Fig. 1a and b). ppBAM verifies these reads are indeed supporting the deletion event by realigning to the deletion allele (Fig. 1c). ppBAM also reveals a large number of ambiguous reads which are attributed to a sequence duplication (GCAGCGC) flanking the deletion, making these reads uninformative for genotyping (Supplementary Fig. S5).

### 3.2 ppBAM genotyping pinpoints a wrong variant call

A wrong variant call is easily flagged by the variant genotyping feature of ppBAM. When visualizing reads alignment for a previously published complex mutation in cancer genome, ppBAM displays only 3 reads supporting the mutation and 106 reads supporting neither reference nor alternative allele, indicating wrong variant call (Supplementary Fig. S6a). Manual correction to the variant increases the number of reads to 102 supporting the alternative allele (Supplementary Fig. S6b) and is further confirmed by realignment (Supplementary Fig. S6c).

## 4 Summary

ppBAM track is an efficient web tool to support read alignment visualization and variant review, using on-the-fly genotyping. Users can use it standalone to access rich BAM resources in NCI GDC or private BAM files using local ppBAM instance, or integrate it into any web-based clinical genomics workflow to assist variant review.

## Supplementary Material

btad300_Supplementary_Data

## Data Availability

All code and data used in this paper are available in the GitHub repository at https://github.com/stjude/proteinpaint.
