# Supplementary material for: ppBAM: ProteinPaint BAM track for read alignment visualization and variant genotyping
Source: Bioinformatics. 2023 May 4;39(5):btad300. doi: 10.1093/bioinformatics/btad300 (PMC10182850; doi:10.1093/bioinformatics/btad300)
Supplement: btad300_Supplementary_Data [file btad300_supplementary_data.zip › Supplementary Figures.docx]

**Supplementary Figures**


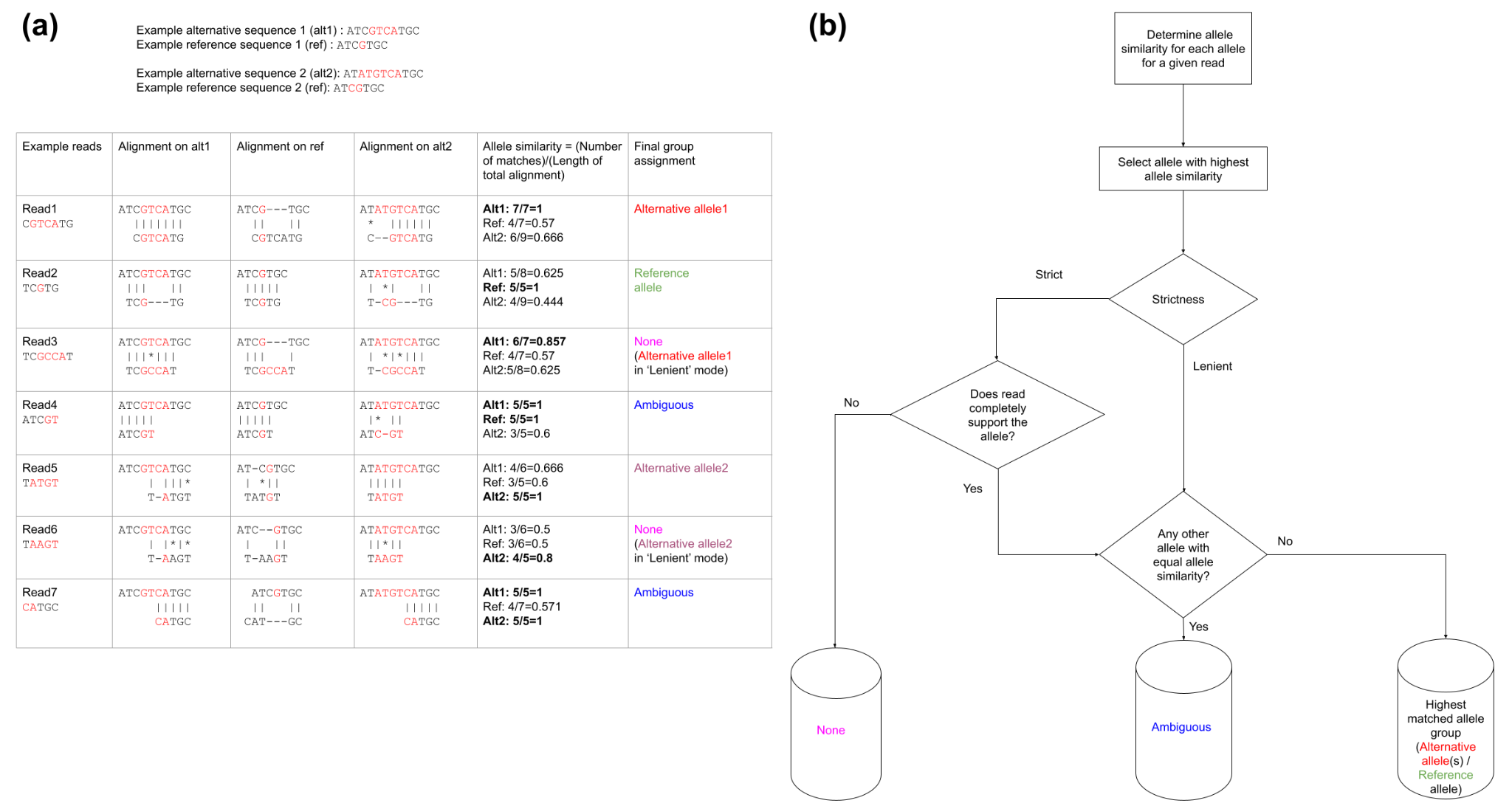


Fig. S1: Using ppBAM genotyping method to classify reads into Reference, Alternative(s), None (neither reference nor alternative allele) and Ambiguous groups based on a variant. (a) An example is described by classifying seven reads against two overlapping variants (G/GTCA) and (CG/ATGTCA). Detailed local alignment by Smith-Waterman method is shown for each read against each alternative and reference alleles (red colored nucleotides represent alternative and reference allele nucleotides). “Allele similarity” is calculated for each read by comparing the identity ratio (number of matched nucleotides/total alignment length) between the read and each of the alleles (highest identity ratio highlighted in bold). (b) A generalized flow chart for classifying reads into (possibly) multiple alternative/reference alleles using “Allele similarity” values and the “Strictness” setting (discussed in Supplementary Tutorial). The “None” group contains reads which do not support neither reference nor alternative allele(s) will be created only when the “Strictness” setting is set to “Strict” (default). Ambiguous group consists of reads that have equal allele similarity to two (or more) alleles.

**
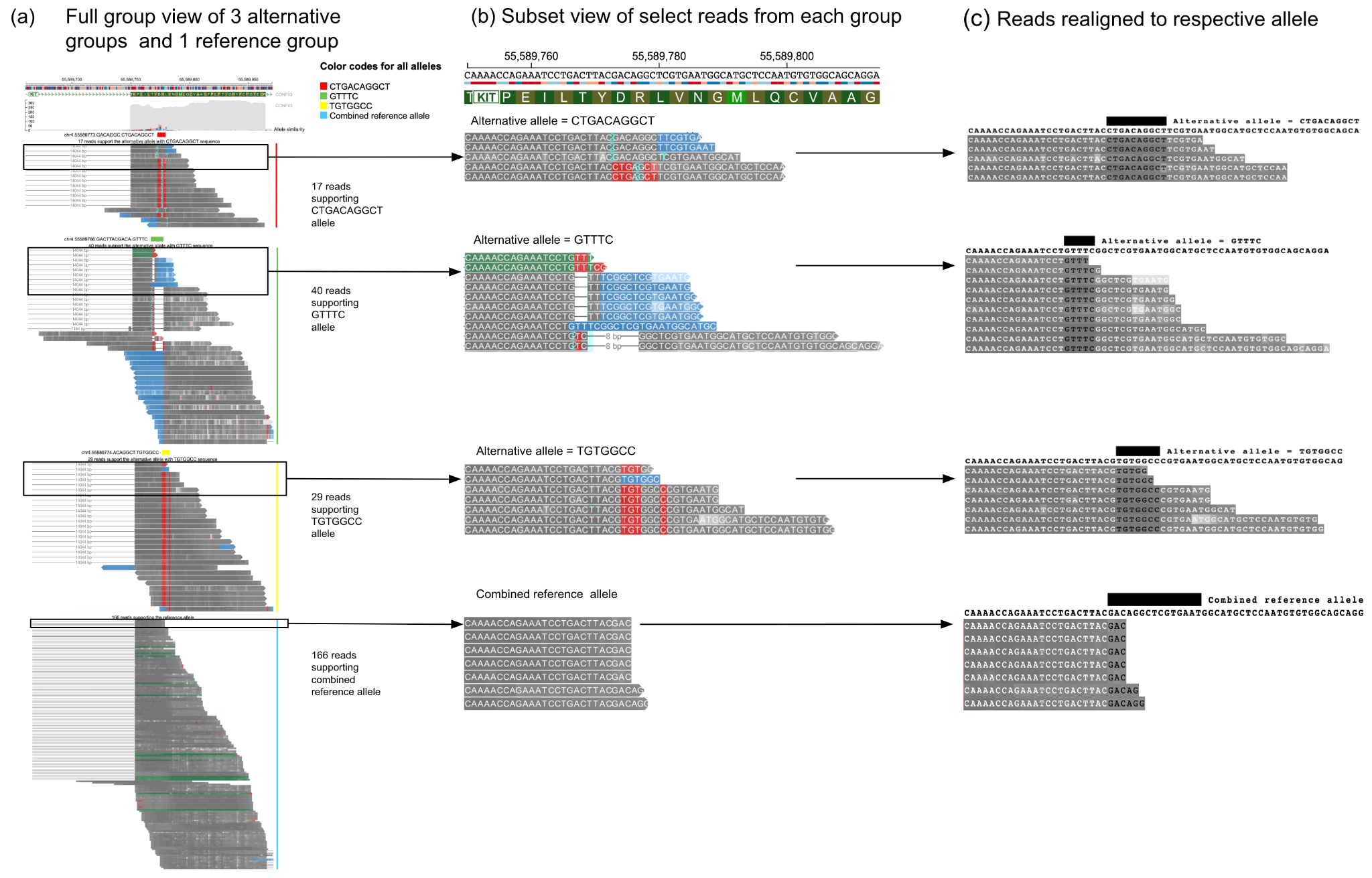
**

Fig. S2: Classification of a [multi-allele variant](https://proteinpaint.stjude.org/?genome=hg19&block=1&position=chr4:55589660-55589870&hlregion=chr4:55589768-55589768&bamfile=multi_allele_variant,proteinpaint_demo/hg19/bam/multi_allele.bam&variant=%7B%22chr%22:%22chr4%22,%20%22variants%22:%5B%7B%22pos%22:55589773,%20%22ref%22:%20%22GACAGGC%22,%20%22alt%22:%20%22CTGACAGGCT%22%7D,%7B%22pos%22:%2055589766,%20%22ref%22:%20%22GACTTACGACA%22,%22alt%22:%22GTTTC%22%7D,%7B%22pos%22:55589774,%22ref%22:%22ACAGGCT%22,%22alt%22:%22TGTGGCC%22%7D%5D%7D&bedjfilterbyname=NM_001385285&strictness=1) using ppBAM. Reads are classified into three alternative alleles with sequences CTGACAGGCT, GTTTC and TGTGGCC in addition to the reference allele, none group and ambiguous group (ambiguous and none groups not shown so as to focus on the unique multi-allele features). Select sets of reads from each group in (a) are zoomed in (b). When the reads from (b) are realigned in (c) to their respective alleles. In case of reads in the reference allele group, the nucleotides in any of the reference alleles are highlighted in (c) (combined reference allele).


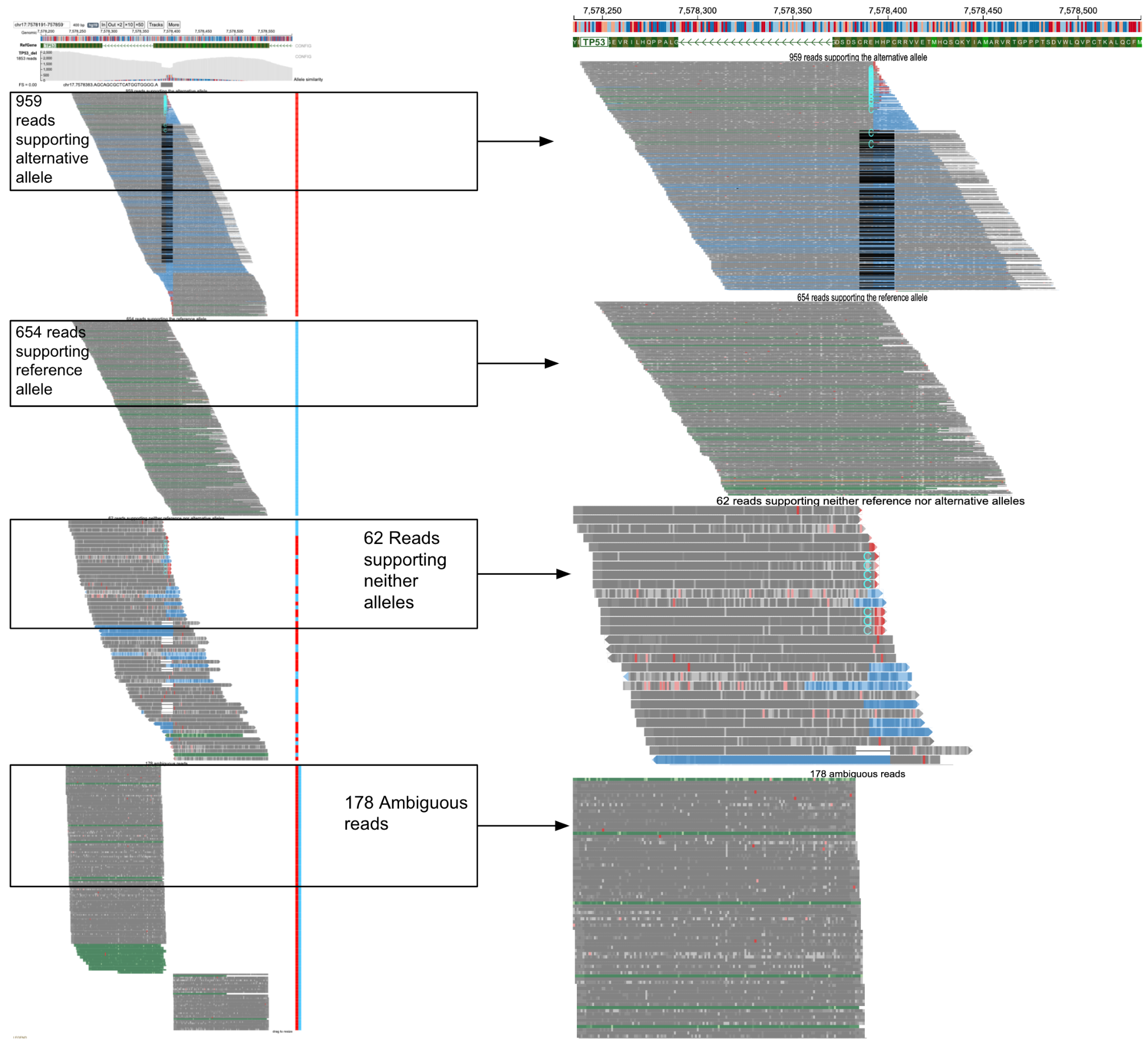


Fig. S3: Deletion in [*TP53* exon](https://proteinpaint.stjude.org/?genome=hg19&block=1&bamfile=TP53_del,proteinpaint_demo/hg19/bam/TP53_del.bam&position=chr17:7578191-7578591&variant=chr17.7578383.AGCAGCGCTCATGGTGGGG.A&bedjfilterbyname=NM_000546). Left: 1853 reads are divided into four groups using the genotyping method, resulting in 959 reads in the alternative group, 654 reads in the reference group, 62 reads in the None group (neither reference nor alternative allele, only in “Strict” mode), and 178 reads in the ambiguous group (with identical match to reference and alternative alleles). Right: enlarged view of the subset of reads in each group. Reads supporting the alternative allele are further examined in Fig. 1. Cause of ambiguous reads is illustrated in Fig. S5.


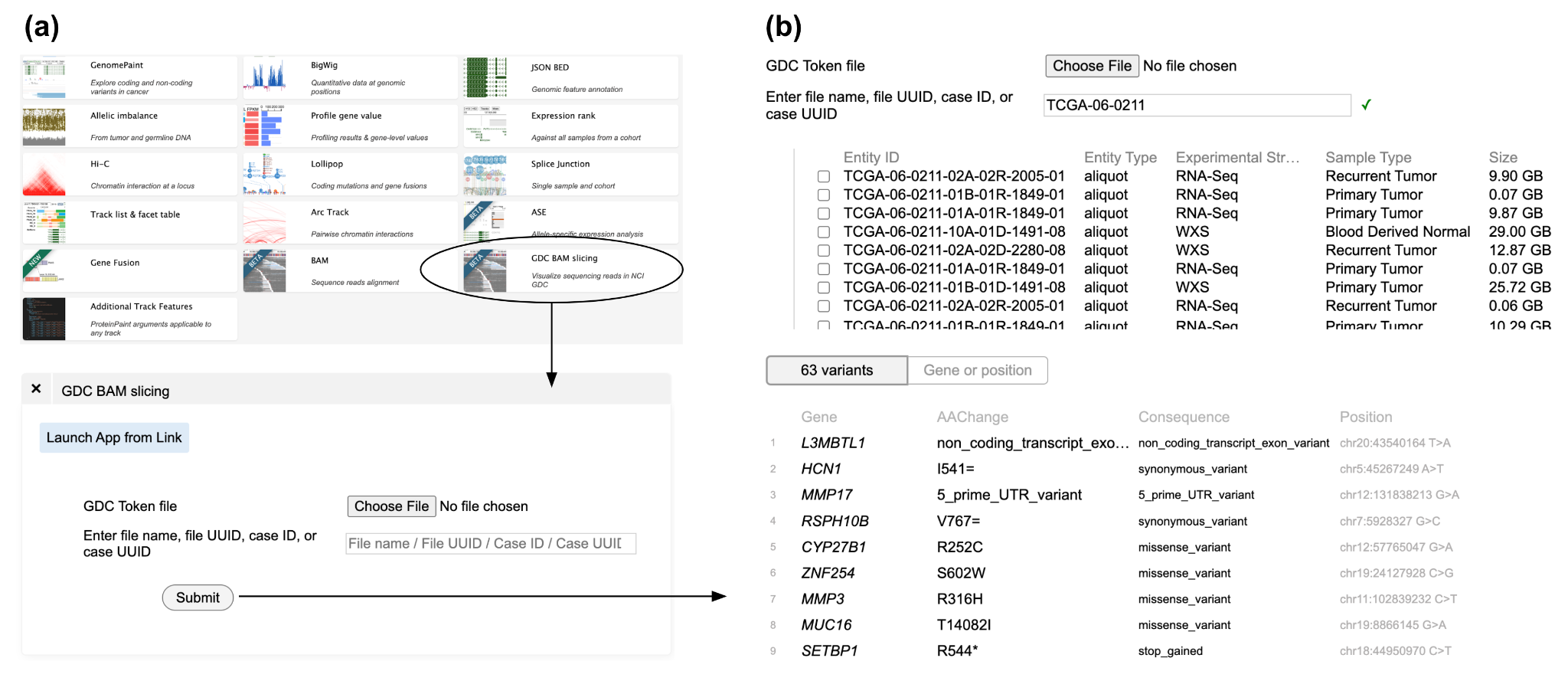


Fig. S4: Using ppBAM to query and visualize BAM files from NCI Genomic Data Commons (GDC). (a) ProteinPaint App Drawer containing the “GDC BAM slicing” card. Click the card to show a panel to upload GDC token and enter GDC patient, sample or file ID. (b) By entering patient ID “TCGA-06-0211”, the panel displays the list of BAM files available for this patient in GDC, showing details including experimental strategy, sample type, etc. Also, a list of somatic coding mutations from the tumor of this patient is retrieved from GDC and can be selected to view on-the-fly genotyping results on a mutation. Alternatively, users can enter a custom genomic region or mutation to view results.


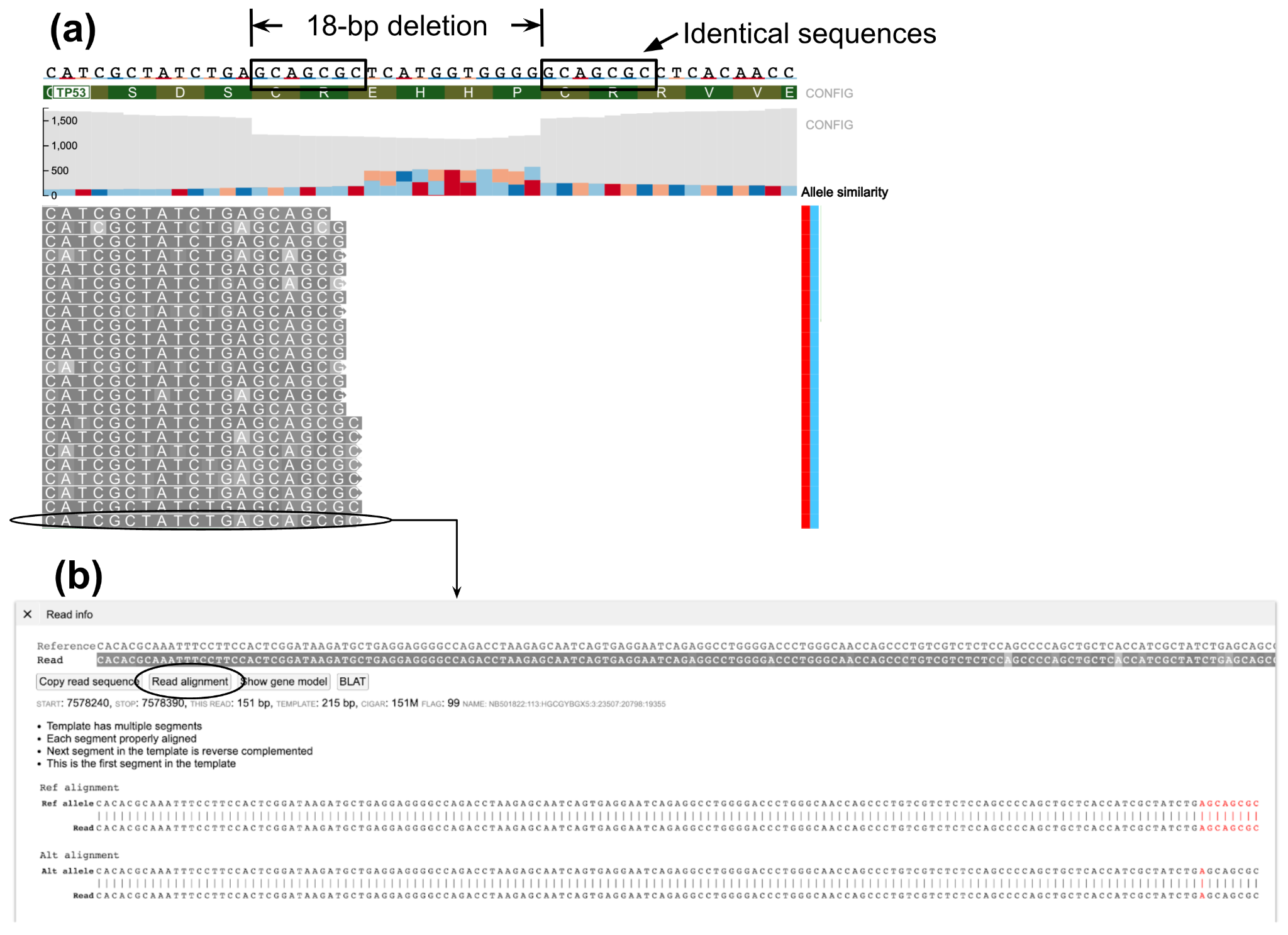


Fig. S5: Ambiguous reads from the *TP53* 18-bp deletion example (Fig. 1) are caused by a 6-bp duplication flanking the deletion. (a) Subset of ambiguous reads with equal similarity to both reference and alternative alleles. Black boxes mark out a 6-bp duplication flanking the deletion region. (b) At the Read Information Panel for the highlighted read in (a), click the “Read alignment” button (oval) to display the pairwise alignments against both reference and alternative alleles, showing equal sequence similarity to both alleles. In the alignments, nucleotides highlighted in red indicate those from reference and alternative alleles.


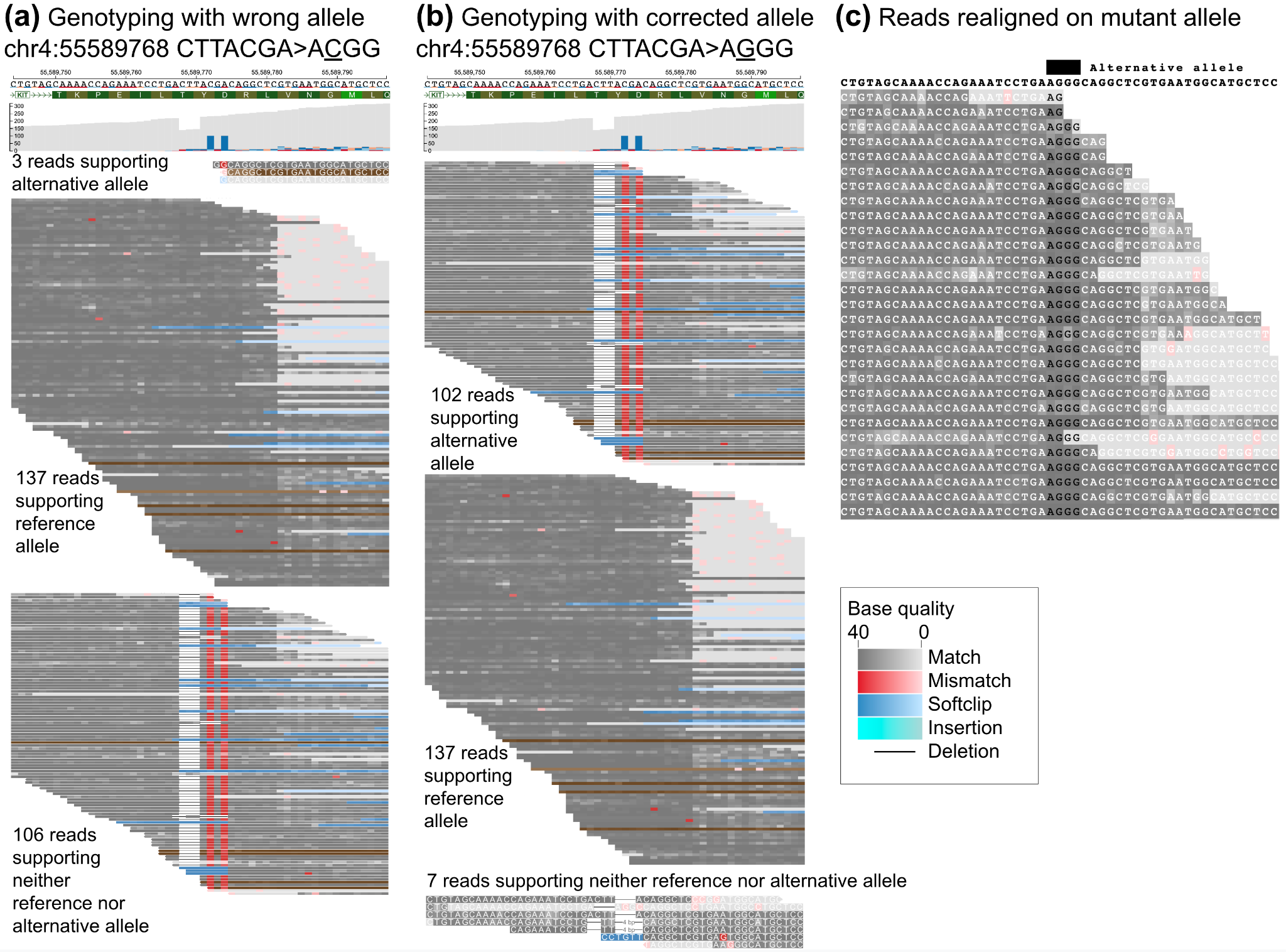


Fig. S6: Illustration of a wrong variant call. (a) [Wrong variant call](https://proteinpaint.stjude.org/?genome=hg19&block=1&bamfile=wrong_indel,proteinpaint_demo/hg19/bam/wrong_indel.bam&position=chr4:55589768-55589770&variant=chr4.55589768.CTTACGA.ACGG&bedjfilterbyname=NM_000222), Reference = CTTACGA; Alternative = ACGG. This variant was reported by a pediatric cancer study (Ma, et. al., 2018, [GenomePaint link to this variant](https://proteinpaint.stjude.org/?genome=hg19&block=1&mds=Pediatric2,svcnv&sample=SJAML040555_D2-PANVGP&position=chr4:55589751-55589805&bedjfilterbyname=NM_000222)). Only 3 reads were found by on-the-fly genotyping to support the alternative allele, indicating wrong variant call. (b) [Correct variant call](https://proteinpaint.stjude.org/?genome=hg19&block=1&bamfile=correct_variant_call,proteinpaint_demo/hg19/bam/wrong_indel.bam&position=chr4:55589768-55589770&variant=chr4.55589768.CTTACGA.AGGG&bedjfilterbyname=NM_000222) by manual inspection of reads. Reference = CTTACGA; Alternative = AGGG. Number of reads supporting alternative allele increases to 102, indicating it is the correct variant call. (c) Realignment panel showing the reads supporting the correct alternative allele (Alternative = AGGG).

**Supplementary Table**

|  | **ProteinPaint BAM track** | **Integrative Genomics Viewer (IGV)** | **BamView** | **BamSnap** | **pileup.js** | **UCSC genome browser BAM track** |
| --- | --- | --- | --- | --- | --- | --- |
| Environment | Web browser | Desktop application and web browser | Desktop application | Desktop application | Web browser | Web browser |
| Web URL | <https://proteinpaint.stjude.org/bam> | <https://software.broadinstitute.org/software/igv/home> | <https://sanger-pathogens.github.io/Artemis/BamView/> | <https://bamsnap.readthedocs.io/en/latest/index.html> | <https://github.com/hammerlab/pileup.js/> | <https://www.genome.ucsc.edu/goldenpath/help/bam.html> |
| Interactive alignment view? | Yes | Yes | Yes | No | Yes | Yes |
| Variant review | Classification of reads into alternative/reference/none/ambiguous groups for both SNV and indels  Also supports multi-allele complex indels. | Limited support. Does not support multi-allele complex indels. | None | None | None | None |
| Realtime realignment of reads supporting alternative allele? | Yes | No | No | No | No | No |
| Support for querying BAM slices from NCI GDC? | Yes | No | No | No | No | No |
| BLAT | Yes | Yes | No | No | No | Yes |

Table S1: Table comparing various features of ppBAM with other BAM file viewers: Integrative Genomics Viewer (IGV), BamView, BamSnap, pileup.js and UCSC genome browser BAM track.
