# Supplementary material for: ppBAM: ProteinPaint BAM track for read alignment visualization and variant genotyping
Source: Bioinformatics. 2023 May 4;39(5):btad300. doi: 10.1093/bioinformatics/btad300 (PMC10182850; doi:10.1093/bioinformatics/btad300)
Supplement: btad300_Supplementary_Data [file btad300_supplementary_data.zip › ProteinPaint BAM track tutorial.docx]

ProteinPaint BAM track (ppBAM) tutorial

[**Introduction 3**](#_nqif2ugigprd)

[**ppBAM source code 3**](#_b5m6a67fabh3)

[**Installing ppBAM and setting up docker image 4**](#_xb80r6o283lx)

[Installation instructions for PP docker image 4](#_sd0jdtm3emet)

[Downloading support files for ppBAM 4](#_oqw90z4y9dgh)

[Create serverconfig.json 4](#_n1q8ckxfp7n2)

[**Input data 5**](#_1032whhxg5nq)

[Viewing local BAM files on user’s workstation using ProteinPaint docker container 5](#_legtwqgizd43)

[Accessing publicly available BAM files using ppBAM 6](#_nfe8wqhbbjal)

[Web URL parameters 6](#_fqqdsdly9ns1)

[Host URL 6](#_uk9elbfa33i9)

[genome=string 6](#_wxsc3ihtptoe)

[block=1 7](#_5nioqdwrq2a7)

[position=string (optional) 7](#_k740vzgxohbg)

[bamfile=string 7](#_8z1nc6t9ik6e)

[bamurl=string 7](#_8a2bha9n67wl)

[bedjfilterbyname=string (optional) 7](#_5hzkevi8868k)

[variant=string (optional) 8](#_8xbzrcxbtx60)

[Single allele syntax 8](#_6jp7ybn48fsf)

[Multi allele syntax 8](#_fzhidfxoli0r)

[Generate local ppBAM weblinks using command-line script 8](#_au776dfpvg49)

[Display BAM files available from NCI GDC 11](#_3jsjcurm6luo)

[**Using ProteinPaint genome browser 12**](#_n93ehy8g604w)

[Current position in genome 12](#_7cjyzjgrjias)

[Reference genome build 12](#_7gbjh07sa9pq)

[Zoom buttons 13](#_194pehb9wdgl)

[Reference genome sequence 13](#_t27sq48ei1z8)

[Gene models 13](#_d8u60ql70ezc)

[**ProteinPaint BAM track features 13**](#_f4qyi7z8ee68)

[Pileup plot 13](#_mdqou3gbd8)

[Read alignment plot 14](#_38dzjdbvti15)

[Rendering of various mutations 14](#_zfnoor7mcu7b)

[Insertion 14](#_unuxk8w8jez4)

[Deletion 15](#_4bhv8dz0u918)

[Substitution (or mismatch) 15](#_h3e8e5pdvgv1)

[Splicing 15](#_d7lmm5kzmfel)

[Zooming the read alignment plot 15](#_4w5ws3tpzf34)

[Horizontal zoom 15](#_cwx0n3yexdjq)

[Overview level 16](#_vcvjdap0uysx)

[Base-pair quality level 16](#_q7tmkzv0ysw7)

[Base-pair resolution level 17](#_x7c2znea1qxu)

[Vertical zoom: examining subset of reads 17](#_qxzh89gdrfte)

[**BAM track configuration panel 18**](#_pk4jzgmhupjq)

[BAM track configuration panel figure 19](#_8dsa4719ln5f)

[Single and paired-end read 19](#_s00wty27zjtv)

[Show/hide read names 21](#_4brxigan6ddg)

[Displaying PCR and optical duplicated reads 21](#_oc2rgco9jzl2)

[Strictness 22](#_2ce7lbdrsxvi)

[**Read information panel 22**](#_7f1mou6qua8k)

[Copy read sequence 22](#_bf4dehk6aj30)

[Show gene models 22](#_oweup990ov6q)

[BLAT 23](#_9r1hd5gbund5)

[QScore 23](#_4gsj4qbv5r6q)

[QStart 23](#_9n6f1vijur96)

[QStop 23](#_ymm6yihg7f8m)

[QAlignLen 23](#_a03dcfvelw2k)

[RChr 23](#_swum2i6r1c3i)

[RStart 24](#_u65whmwpm6j1)

[RStop 24](#_ofqiv5b81yxc)

[RAlignLen 24](#_p13zkne8czft)

[Read details 24](#_5c9h1uj4hi3l)

[CHR 24](#_vsowe0gegyaj)

[START 24](#_llqrhdhdofi)

[STOP 24](#_86o6g288vbn1)

[READ LENGTH 24](#_vd18yzvt4dli)

[TEMPLATE LENGTH 24](#_r0lvlyslh9ve)

[CIGAR 24](#_n95uw93txurp)

[FLAG 24](#_ggyi4p63lfbk)

[NAME 25](#_hble3mbn4u7v)

[Color coding of reads 25](#_m2mkufdeqgt4)

[Gray 26](#_z37cikuclyyc)

[Blue 27](#_bkq91auo81mr)

[Brown 27](#_mjmjzl9v5jzq)

[Green 28](#_m9o6fj3vyszc)

[Pink 28](#_mckp9b26iuws)

[Orange 29](#_183vhdcsv3nh)

[**Variant mode 29**](#_suyvbwrw760i)

[Read classification into four (or more) groups: Alternative, Reference, None and Ambiguous 30](#_wozvoeejv04y)

[Ambiguous reads 31](#_rdmthgg2ezwa)

[Fisher-strand analysis to check for strand bias in variants 32](#_4hebafka9zrf)

[Strictness in on-the-fly genotyping 33](#_h2q7c289qfvv)

[Realignment using Clustal Omega 34](#_heeusjhsujl6)

[Display of read alignment with respect to both reference and alternative allele 34](#_79s25evdepax)

[Classification of multi-allele variants 35](#_rxqat4my59rq)

# Introduction

ProteinPaint BAM track (ppBAM) is a web visualization tool to visualize read alignments from a BAM file. Given a variant (i.e. Chromosome number, Position, Reference Allele and Alternative Allele) it can classify reads supporting the reference and alternative allele into separate groups.

# ppBAM source code

Source code is available for non-commercial use and can be accessed from Github using this [link](https://github.com/stjude/proteinpaint). ppBAM is part of an integrated set of tools ([ProteinPaint](https://proteinpaint.stjude.org/) (PP)) for [querying and visualizing genomic data](https://www.ncbi.nlm.nih.gov/pmc/articles/PMC7884056/). Therefore, this repository hosts the source code for all these tracks along with that of ppBAM.

Source code files pertaining to ppBAM:

1) [client/src/block.tk.bam.js](https://github.com/stjude/proteinpaint/blob/master/client/src/block.tk.bam.js) - This contains code that runs within the user’s browser (client-side code). This makes a request to server side providing the genomic location (and variant) so as to query that particular region from the BAM file

2) [server/src/bam.js](https://github.com/stjude/proteinpaint/blob/master/server/src/bam.js) - Server side code which queries the BAM files for reads in the genomic location passed by the user’s browser. It also renders the image and passes it back to the user’s browser.

3) [server/src/bam.kmer.indel.js](https://github.com/stjude/proteinpaint/blob/master/server/src/bam.kmer.indel.js) - Server side code that interacts with the SNV/indel classification pipeline (written in Rust programming language). It passes the [variant](#_suyvbwrw760i) information to the SNV/indel classification pipeline and collects the classification of reads.

4) [rust/src/indel.rs](https://github.com/stjude/proteinpaint/blob/master/rust/src/indel.rs) - Implementation of the [SNV/indel classification pipeline in Rust](#_wozvoeejv04y).

5) [rust/src/align.rs](https://github.com/stjude/proteinpaint/blob/master/rust/src/align.rs) - Generates the read alignment to various alleles when the “[Read Alignment](#_79s25evdepax)” button is clicked through the [read information panel](#_7f1mou6qua8k).

6) [rust/src/realign.rs](https://github.com/stjude/proteinpaint/blob/master/rust/src/realign.rs) - Contains common functions used by both indel.rs and align.rs. For example, function to align a read to a particular allele.

7) [rust/src/stats_functions.rs](https://github.com/stjude/proteinpaint/blob/master/rust/src/stats_functions.rs) - Contains the fisher’s exact test function for carrying out [fisher-strand analysis to check for strand bias](#_4hebafka9zrf).

# Installing ppBAM and setting up docker image

## Installation instructions for PP docker image

ProteinPaint has a substantial number of software dependencies. Therefore, it is best run locally using the PP docker image. The user may download the pre-built docker image or generate a docker image directly from source code to get the latest version of PP. For this the user needs to download [Docker](https://www.docker.com/) or [Docker Desktop](https://www.docker.com/products/docker-desktop/).

For installing ppBAM using the docker image, please refer to this [link](https://github.com/stjude/proteinpaint/blob/master/container/README.md) for detailed instructions.

## Downloading support files for ppBAM

Other than the docker image, data files are needed for the reference genomes being supported by the local ppBAM instance. These can be downloaded using the script [public-support-files-download.sh](https://github.com/stjude/proteinpaint/blob/master/utils/public-support-files-download.sh). Create a new directory (for e.g. ppData) which will store all the data files for the ppBAM local docker instance.

| $ mkdir ppData && cd ppData  $ wget <https://raw.githubusercontent.com/stjude/proteinpaint/master/utils/public-support-files-download.sh>  $ chmod a+x public-support-files-download.sh  $ ./public-support-files-download.sh |
| --- |

This script may take 10-15 mins for downloading reference genome data files depending on internet speed.

## Create serverconfig.json

serverconfig.json file is the configuration file needed so as to pass the path of all the data files needed to run ppBAM. As an example, the serverconfig.json file is shown for a local PP installation supporting the hg19 and hg38 reference genome builds.

| {  "debugmode": true,  "defaultgenome": "hg19",  "features": {},  "genomes": [  {  "name": "hg19",  "file": "./genome/hg19.js",  "species": "human"  },  {  "name": "hg38",  "file": "./genome/hg38.js",  "species": "human"  }  ],  "url": "http://localhost:3456",  "tpmasterdir": "/path/to/ppData",  "backend_only": false  } |
| --- |

This file must be placed in the directory from where the ppBAM docker container is being run. The “url” field must point to any port (in this example 3456) that is open. The “tpmasterdir” field must contain the absolute path to the [directory where data files for the local ppBAM instance are stored](#_oqw90z4y9dgh) (in this example ppData).

# Input data

## Viewing local BAM files on user’s workstation using ProteinPaint docker container

For viewing local BAM files, the syntax as defined in the section [web URL parameters](#_fqqdsdly9ns1). Path to BAM file must be from the root data folder. For e.g. /path/to/ppData/proteinpaint_demo/hg19/bam/TP53_del.bam will become proteinpaint_demo/hg19/bam/TP53_del.bam in the web URL. The final URL will be

<http://localhost:3456/?genome=hg19&block=1&bamfile=TP53_del,proteinpaint_demo/hg19/bam/TP53_del.bam&position=chr17:7578191-7578591&variant=chr17.7578383.AGCAGCGCTCATGGTGGGG.A&bedjfilterbyname=NM_000546>

Multi-allele variant example

[http://localhost:3456/?genome=hg19&block=1&position=chr4:55589660-55589870&bamfile=multi_allele_variant,proteinpaint_demo/hg19/bam/multi_allele.bam&variant={"chr":"chr4", "variants":[{"pos":55589773, "ref": "GACAGGC", "alt": "CTGACAGGCT"},{"pos": 55589766, "ref": "GACTTACGACA","alt":"GTTTC"},{"pos":55589774,"ref":"ACAGGCT","alt":"TGTGGCC"}]}&bedjfilterbyname=NM_001385285](http://localhost:3456/?genome=hg19&block=1&position=chr4:55589660-55589870&bamfile=multi_allele_variant,proteinpaint_demo/hg19/bam/multi_allele.bam&variant=%7B)

## Accessing publicly available BAM files using ppBAM

The user can also choose to view read alignment from a BAM file available through a public URL. This can be done by specifying the weblink URL in the bamurl field in [web URL parameters](#_fqqdsdly9ns1). In the example below, reads from the breast cancer cell line HCC1143BL are being visualized.

<https://proteinpaint.stjude.org/?genome=hg19&block=1&bamurl=HCC1143BL,http://genomedata.org/gen-viz-workshop/IGV/HCC1143.normal.21.19M-20M.bam&position=chr21:18999750-19003031>

If using local PP docker image with port: 3456

<http://localhost:3456/?genome=hg19&block=1&bamurl=HCC1143BL,http://genomedata.org/gen-viz-workshop/IGV/HCC1143.normal.21.19M-20M.bam&position=chr21:18999750-19003031>

## Web URL parameters

Example URL to launch a BAM file in official ProteinPaint server (or using local ProteinPaint instance) using ppBAM track:

<https://proteinpaint.stjude.org/?genome=hg19&block=1&bamfile=TP53_del,proteinpaint_demo/hg19/bam/TP53_del.bam&position=chr17:7578191-7578591&variant=chr17.7578383.AGCAGCGCTCATGGTGGGG.A&bedjfilterbyname=NM_000546>

### Host URL

<https://proteinpaint.stjude.org> (or localhost:3456 of PP docker image)

### genome=string

The genome build against which the DNA/RNA sequence reads has been mapped. Genome builds currently supported are the following:

Human genome builds supported:

hg19

hg38

Mouse genome builds supported:

mm9

mm10

Zebrafish genome build supported:

danRer10

Fruit fly genome builds supported:

dm3

dm6

Chicken genome builds supported:

galGal5

galGal6

Rat genome builds supported:

rn6

### block=1

This parameter displays the [gene models](#_d8u60ql70ezc). Set block=1 (boolean flag) to display gene models.

### position=string (optional)

Position range to be visualized in the format {chr}:{start position}-{end position}.

### bamfile=string

Name of the BAM file to be visualized, path to BAM file relative to root data directory.

### bamurl=string

Name of the BAM file to be visualized, public http link to the BAM file.

### bedjfilterbyname=string (optional)

An optional feature which allows display of only one single gene model when multiple gene models are available for the display region.

Example:

The given example below shows Exon 5 of *TP53* from the BAM file “TP53_del.bam” at the location “proteinpaint_demo/hg19/bam/”. This is visualized using the human genome build hg19 at the coordinates “chr17:7578191-7578591”.

<https://proteinpaint.stjude.org/?genome=hg19&block=1&bamfile=TP53_del,proteinpaint_demo/hg19/bam/TP53_del.bam&position=chr17:7578191-7578591&bedjfilterbyname=NM_000546>

### variant=string (optional)

An optional parameter that invokes the [variant-typing mode](#_suyvbwrw760i). When specified, reads covering the variant region are classified into Reference, Alternative, None (neither reference nor alternative allele) and Ambiguous groups.

#### Single allele syntax

{chr}.{position}.{reference allele}.{alternative allele}.

#### Multi allele syntax

[Multi allele variants](#_rxqat4my59rq) are represented as a JSON string in the variant field.

{"chr":Chromosome_name, "variants":[{"pos":pos1, "ref": ref1, "alt": alt1},{"pos": pos2, "ref": ref2,"alt":alt2}]}

where

pos1 - position of 1st variant

ref1 - reference allele of 1st variant

alt1 - alternative allele of 1st variant

pos2 - position of 2nd variant

ref2 - reference allele of 2nd variant

alt2 - alternative allele of 2nd variant

For example, a multi-allele variant with 3 different variants on chromosome “chr4”: 55589773.GACAGGC.CTGACAGGCT, 55589766.GACTTACGACA.GTTTC and 55589774.ACAGGCT.TGTGGCC will be represented in JSON format as the following:

{"chr":"chr4", "variants":[{"pos":55589773, "ref": "GACAGGC", "alt": "CTGACAGGCT"},{"pos": 55589766,"ref":"GACTTACGACA","alt":"GTTTC"},{"pos":55589774,"ref":"ACAGGCT","alt":"TGTGGCC"}]}

## Generate local ppBAM weblinks using command-line script

For convenience, a python command line script ([ppBAMlinks_generator.py](https://github.com/stjude/proteinpaint/blob/master/utils/ppBAMlinks_generator.py)) has been provided which can extract variants from a single-sample VCF file and generate a ppBAM weburl for each variant entry in the VCF file. It also parses adjacent rows of the VCF file to check if there is coordinate overlap between SNV/indels. In case an overlap is found, a multi-allele view is generated by inserting all the overlapped variants into a single [multi-allele JSON](#_fzhidfxoli0r) string as shown below.

In the example below, the variant entries from a single-sample VCF file ([test4.vcf](https://proteinpaint.stjude.org/ppdemo/test4.vcf)) are shown below

| #CHROM POS ID REF ALT QUAL FILTER INFO FORMAT NA00001  chr2 14370 . G A 29 PASS . GT 1\|0  chr2 17330 . T A 3 PASS . GT 1\|0  chr2 1110696 . A G,T 67 PASS . GT 1\|0  chr2 1234567 . GTCT G,GTACT 50 PASS . GT 0\|1  chr4 55589766 . GACTTACGACA GTTTC 3 PASS . GT 1\|0  chr4 55589773 . GACAGGC CTGACAGGCT 29 PASS . GT 1\|0  chr4 55589774 . ACAGGCT TGTGGCC 67 PASS . GT 1\|0  chr4 55589790 . A G 30 PASS . GT 1\|0 |
| --- |

Creating a virtual environment and installing PyVCF3 dependency

| # Create a python virtual environment  $ python3 -m venv vcf_parse  # Activate the virtual environment  $ source vcf_parse/bin/activate  # Install PyVCF3 module  $ pip3 install PyVCF3  # Download ppBAMlinks_generator.py script  $ wget https://raw.githubusercontent.com/stjude/proteinpaint/master/utils/ppBAMlinks_generator.py  # Download test vcf file  $ wget https://proteinpaint.stjude.org/ppdemo/test4.vcf |
| --- |

Various options available ppBAMlinks_generator.py

| $ python3 ppBAMlinks_generator.py -h  usage: ppBAMlinks_generator [-h] --VCF VCF --BAM BAM [--IP IP] [--out OUT]  [--reference_genome REFERENCE_GENOME] [--view_range VIEW_RANGE]  [--sample_name SAMPLE_NAME]  Generates weblinks in a csv file for each variant from the given VCF file  options:  -h, --help show this help message and exit  --VCF VCF path to input single-sample vcf file is required  --BAM BAM path to input BAM file is required  --IP IP IP address of proteinpaint server  --out OUT Output csv file  --reference_genome REFERENCE_GENOME  Reference genome (e.g hg19,hg38)  --view_range VIEW_RANGE  View range from the variant position  --sample_name SAMPLE_NAME  Name of sample  Text at the bottom of help |
| --- |

Download test VCF file

| $ wget https://proteinpaint.stjude.org/ppdemo/test4.vcf |
| --- |

Syntax for generating ppBAM weblinks

| $ python3 ppBAMlinks_generator.py --VCF test4.vcf --BAM test.bam |
| --- |

All other arguments other than VCF and BAM (shown above) are optional.

The weblinks generated are shown below

| localhost:3000/?genome=hg19&block=1&position=chr2:14070-14670&bamfile=test,test.bam&variant=chr2.14370.G.A  localhost:3000/?genome=hg19&block=1&position=chr2:17030-17630&bamfile=test,test.bam&variant=chr2.17330.T.A  localhost:3000/?genome=hg19&block=1&position=chr2:17030-17630&bamfile=test,test.bam&variant={"chr":"chr2", "variants":[{"pos":1110696, "ref":"A","alt":"G"},{"pos":1110696, "ref":"A","alt":"T"}]}  localhost:3000/?genome=hg19&block=1&position=chr2:17030-17630&bamfile=test,test.bam&variant={"chr":"chr2", "variants":[{"pos":1234567, "ref":"GTCT","alt":"G"},{"pos":1234567, "ref":"GTCT","alt":"GTACT"}]}  localhost:3000/?genome=hg19&block=1&position=chr4:55589474-55590074&bamfile=test,test.bam&variant={"chr":"chr4", "variants":[{"pos":55589766, "ref":"GACTTACGACA","alt":"GTTTC"},{"pos":55589773, "ref":"GACAGGC","alt":"CTGACAGGCT"},{"pos":55589774, "ref":"ACAGGCT","alt":"TGTGGCC"}]}  localhost:3000/?genome=hg19&block=1&position=chr4:55589490-55590090&bamfile=test,test.bam&variant=chr4.55589790.A.G |
| --- |

Each of the web links generated above show the region for each variant in the vcf file. The first two lines in the output consist of single-allele variants. In the case of the third and fourth lines, both represent a multi-allele variant as its corresponding VCF entry consists of two alternate alleles. The fifth line is also a multi-allele variant because the variants in the corresponding VCF file overlap with each other. The sixth line is a single-allele variant.

NOTE: The 5th entry in the output corresponds to the [multi-allele example](https://proteinpaint.stjude.org/?genome=hg19&block=1&position=chr4:55589660-55589870&hlregion=chr4:55589768-55589768&bamfile=multi_allele_variant,proteinpaint_demo/hg19/bam/multi_allele.bam&variant=%7B%22chr%22:%22chr4%22,%20%22variants%22:%5B%7B%22pos%22:55589773,%20%22ref%22:%20%22GACAGGC%22,%20%22alt%22:%20%22CTGACAGGCT%22%7D,%7B%22pos%22:%2055589766,%20%22ref%22:%20%22GACTTACGACA%22,%22alt%22:%22GTTTC%22%7D,%7B%22pos%22:55589774,%22ref%22:%22ACAGGCT%22,%22alt%22:%22TGTGGCC%22%7D%5D%7D&bedjfilterbyname=NM_001385285&strictness=1).

## Display BAM files available from NCI GDC

The NCI Genomic Data Commons (GDC, <https://portal.gdc.cancer.gov/>) contains vast amounts of sequencing data from cancer patients. It also allows downloading BAM file slices using GDC token. ppBAM supports the GDC BAM slicing API and automates the process beginning with sample search, to user authentication, gene/variant selection, BAM slicing, and finally visualization.

A GDC token is required for user authentication. To obtain a GDC token, the user can sign up for an eRA account. Details for setting up an eRA account are described [here](https://gdc.cancer.gov/access-data/obtaining-access-controlled-data).

Figure below outlines the steps for accessing BAM files from GDC.


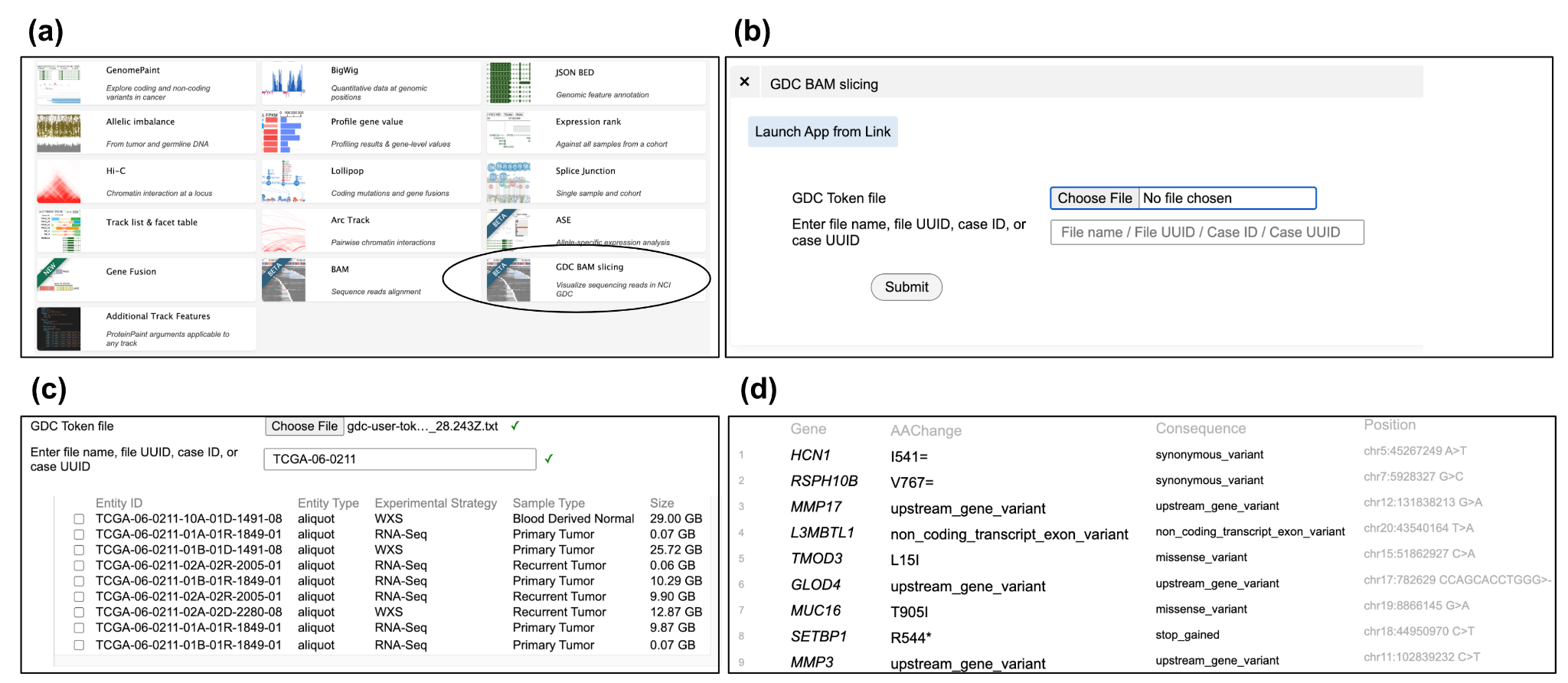


Go to <https://proteinpaint.stjude.org> and click on the “GDC BAM slicing” card. Upload the GDC token file, and enter the GDC case ID. On providing this information, all BAM files associated with this case will be displayed along with the somatic variants. User can select a BAM file from the case (if there are multiple) and a somatic mutation, and click “Submit” to view the BAM slices.

Alternatively, the user may enter a variant, gene or SNP as shown below.


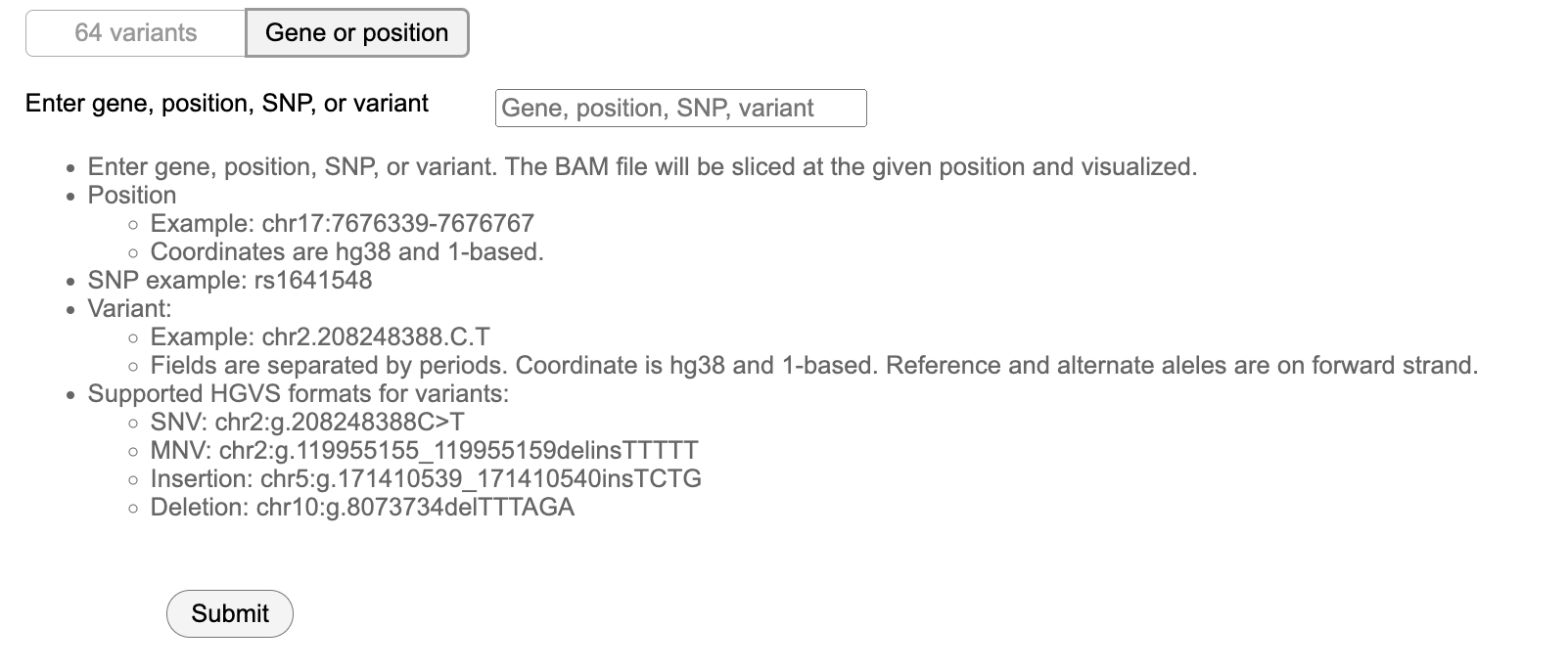


# Using ProteinPaint genome browser

**
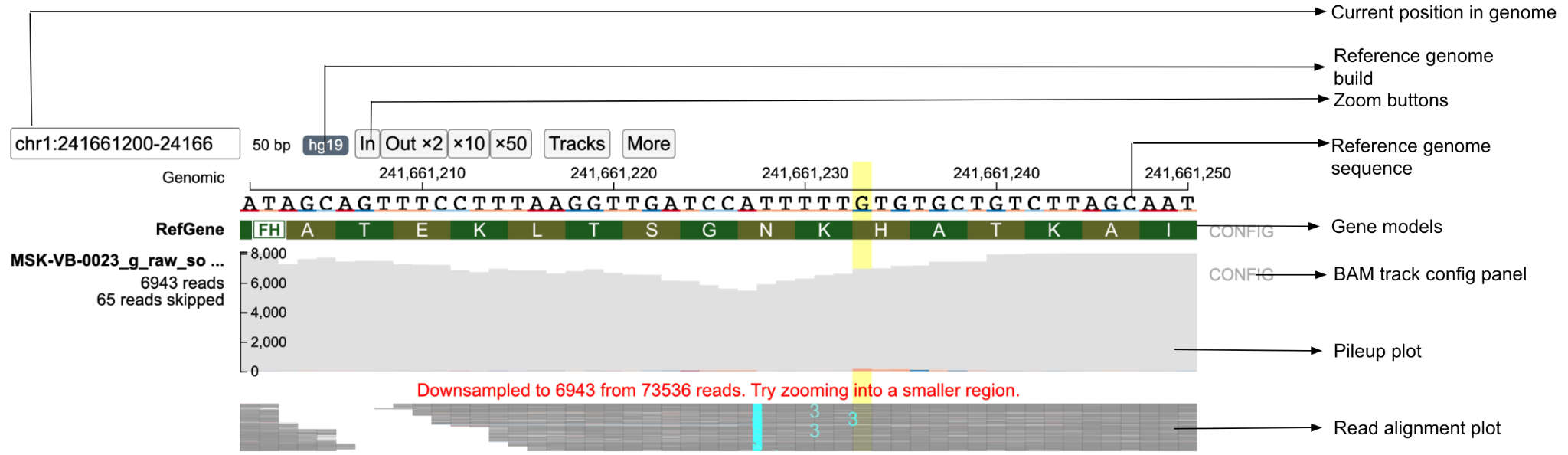
**

Various fields labeled in above figure are described below:

## Current position in genome

It displays the coordinates of the region currently displayed on the screen. It initially shows the coordinates specified in the URL. On pan/zoom by the user this region displays the updated coordinates of the view region.

## Reference genome build

Genome build specified by the user that was used for mapping the reads.

## Zoom buttons

The user can zoom in/out of the current view by clicking the “In” (zoom in) or “Out x2” (zoom out) buttons. By clicking on the x10 and x50 button the user can zoom out 10 and 50-fold respectively. Alternatively, the user may choose to zoom into a smaller region by dragging on the genomic ruler (a) to zoom into the selected region (b) as shown below.


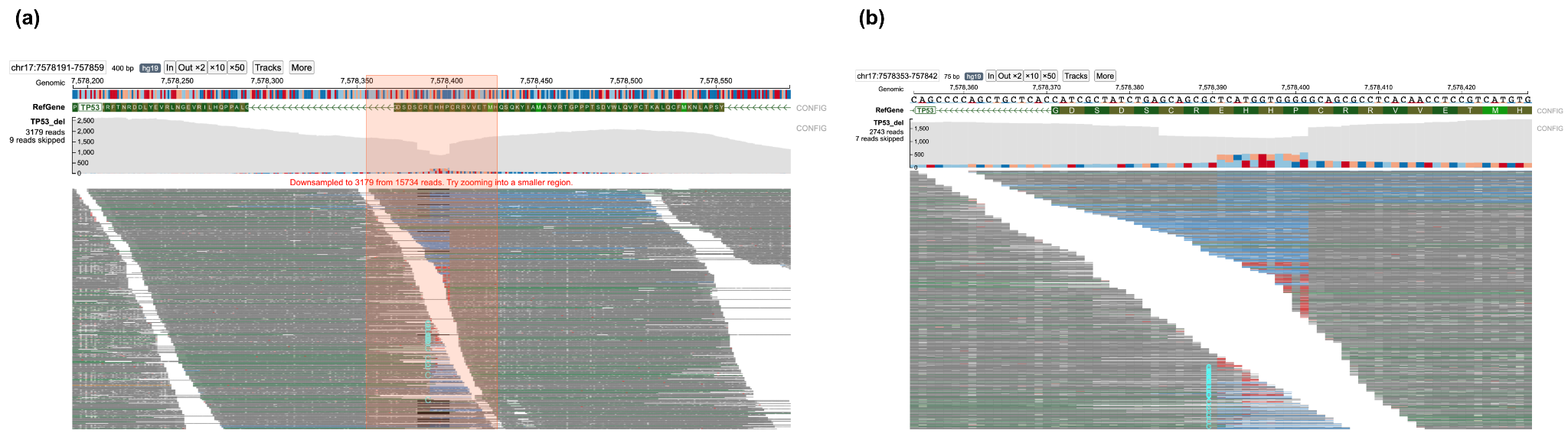


## Reference genome sequence

Displays the reference genome build against which the reads have been aligned. Regions of the genome marked to contain repetitive sequences (by RepeatMasker) are represented by lower-case letters and non-repetitive sequences are represented by upper-case letters.

## Gene models

This row displays gene model structure from the view range. When zoomed into a coding exon, the letters correspond to the 1-letter amino acid code for each amino acid and are placed under its corresponding 3-letter nucleotide codon under the reference genome sequence. The arrows describe the orientation of the strand of the gene model being displayed (right arrow for forward strand and left arrow for reverse strand).

# ProteinPaint BAM track features

## Pileup plot

**
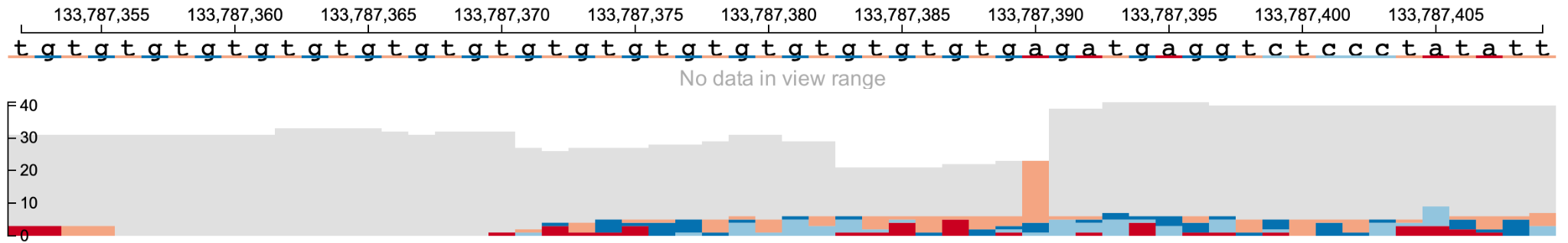
**

The plot shows the total read depth at each nucleotide position of the region being displayed.

Color codes of bars representing various possibilities:

Gray - Reference allele nucleotides

Blue - Soft clipped nucleotides

Mismatches:

- nucleotide “A” - Red (color code: #ca0020)
- nucleotide “T” - Orange (color code: #f4a582)
- nucleotide “C” - Light blue (color code: #92c5de)
- nucleotide “G” - Dark blue (color code: #0571b0)

## Read alignment plot


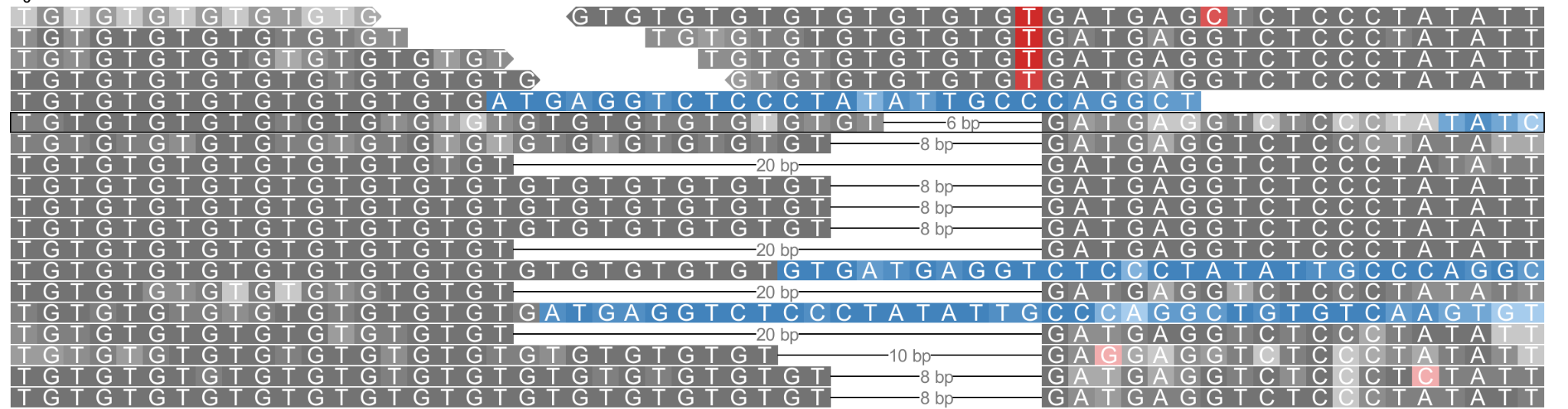


This contains the main read alignment plot of the reads from the BAM file.

### Rendering of various mutations

#### Insertion

In case of a single nucleotide insertion, the alphabet representing the nucleotide (A/T/C/G) is displayed between the two reference nucleotides in cyan color.


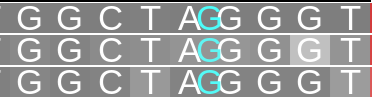


Darkness of the inserted nucleotide is determined by the base quality, as an example below of an inserted T with low quality.


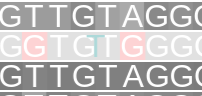


If more than one nucleotide is inserted, a number is printed between the two reference nucleotides indicating the number of inserted nucleotides. The text color is full cyan and does not account for the quality of inserted bases. Showing below is a read with two insertions, first with 2 bases, and second with T.


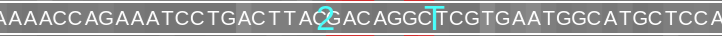


On clicking this read, the [read information panel](#_7f1mou6qua8k) is displayed where the complete inserted nucleotide sequence is shown in cyan color.


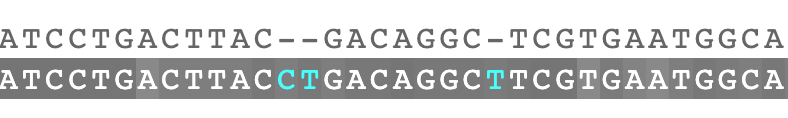


#### Deletion

A black line represents the span of deleted bases.

#####
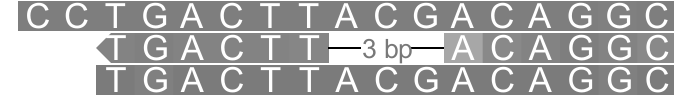


#### Substitution (or mismatch)

In case of substitutions (or mismatches), the substituted nucleotide (“A”) is highlighted in red background, with the shade of red scaled by base quality.


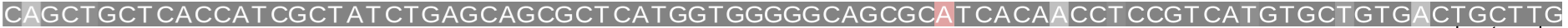


#### Splicing

In case of splicing, the different fragments of a read separated due to splicing are joined by a gray line as shown below. In the example below, the reads contain spliced fragments that are separated by a 1915bp intron.


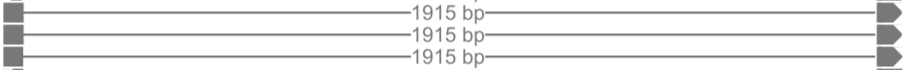


### Zooming the read alignment plot

The rendering of the reads depends upon the zoom level (horizontal zoom) chosen by the user and the number of reads mapped at the display region (vertical zoom).

#### Horizontal zoom

The BAM track has three levels of horizontal zoom:

##### Overview level

This is the completely zoomed out mode (shown below). [At this resolution](https://proteinpaint.stjude.org/?genome=hg19&block=1&bamfile=TP53_del,proteinpaint_demo/hg19/bam/TP53_del.bam&position=chr17:7575308-7580395&bedjfilterbyname=NM_000546), base-pair quality of each nucleotide in each read is not displayed as each read occupies a very small area on the screen. Also the [reference sequence](#_t27sq48ei1z8) at the top is not displayed. Only reads which contain big insertions/deletions/softclips or are discordant are represented by their respective colors (see [color codes of various reads](#_m2mkufdeqgt4)).


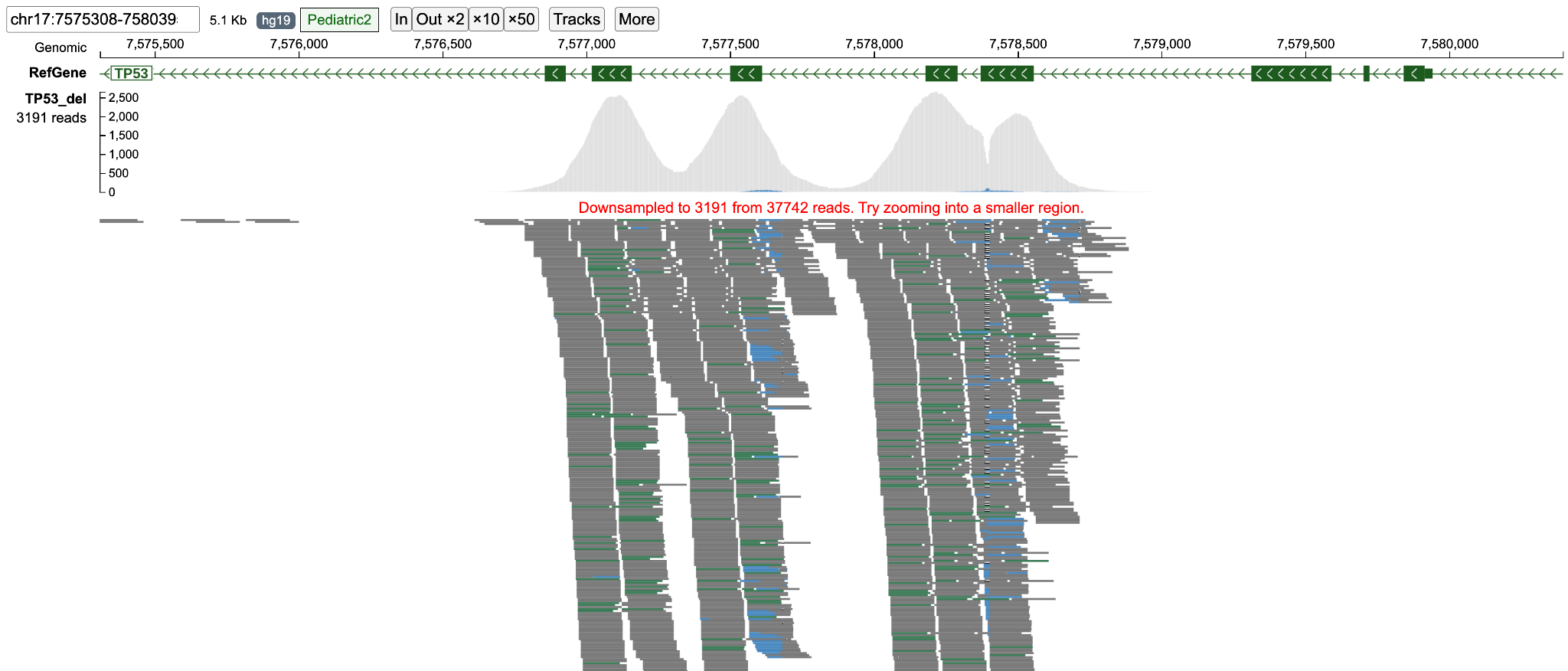


##### Base-pair quality level

[At this level of zoom](https://proteinpaint.stjude.org/?genome=hg19&block=1&bamfile=TP53_del,proteinpaint_demo/hg19/bam/TP53_del.bam&position=chr17:7577215-7578486&bedjfilterbyname=NM_000546) (shown below), in addition to color codes of reads, the phred base pair quality score of each nucleotide in the read is also displayed. Poor base-pair quality of nucleotides is represented by lighter shades of the respective color and darker shades represent high base-pair quality. For example, dark gray color represents a higher quality nucleotide in a properly mapped read than light gray which represents poor base-pair quality.


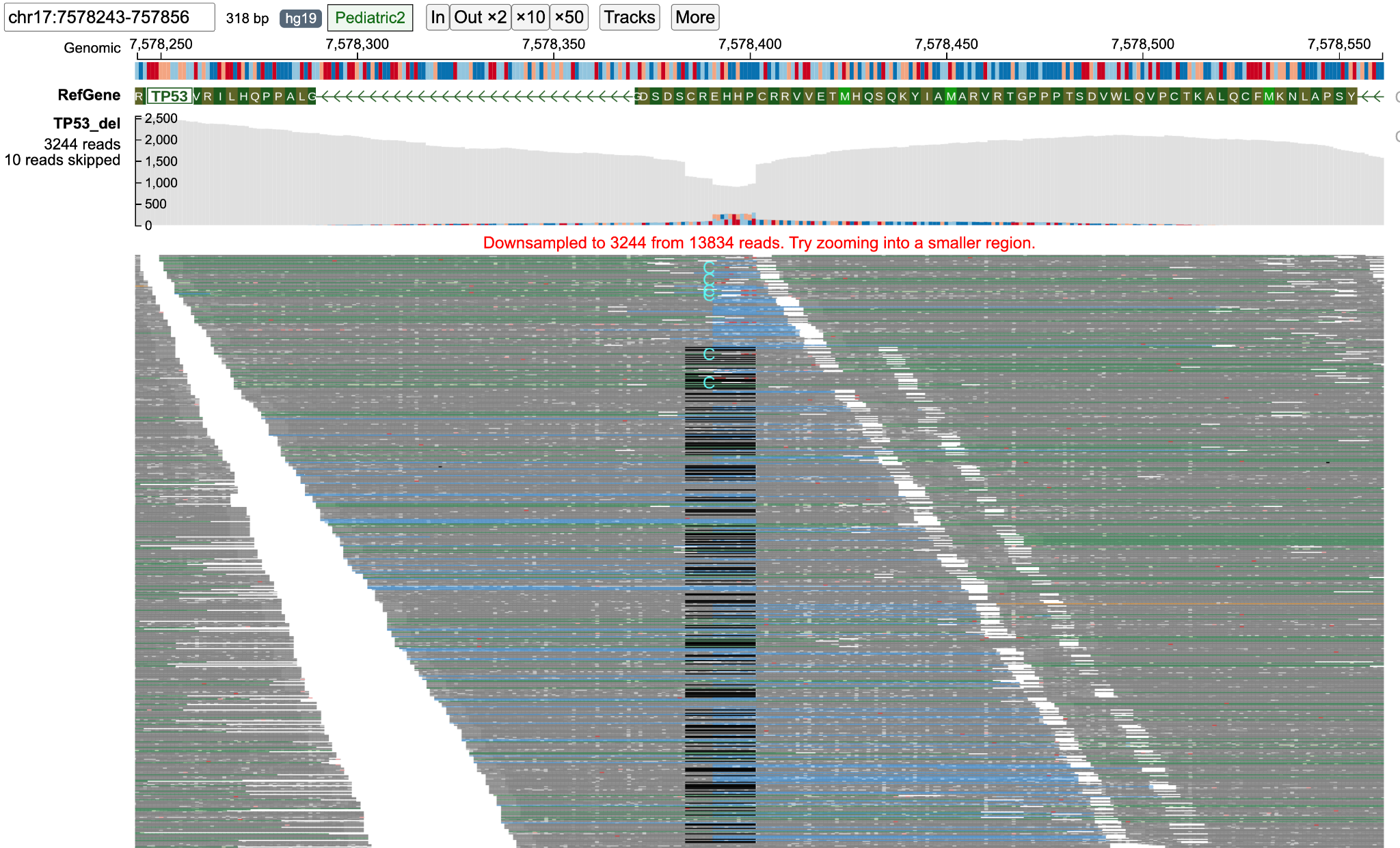


##### Base-pair resolution level

[At this resolution](https://proteinpaint.stjude.org/?genome=hg19&block=1&bamfile=TP53_del,proteinpaint_demo/hg19/bam/TP53_del.wrongbp.bam&position=chr17:7578371-7578417&bedjfilterbyname=NM_000546), all information including the read sequence of each read is displayed along with reference genome nucleotides at the top. For simplicity (as discussed later under [vertical zoom](#_qxzh89gdrfte)), only a few reads are shown in the figure below.


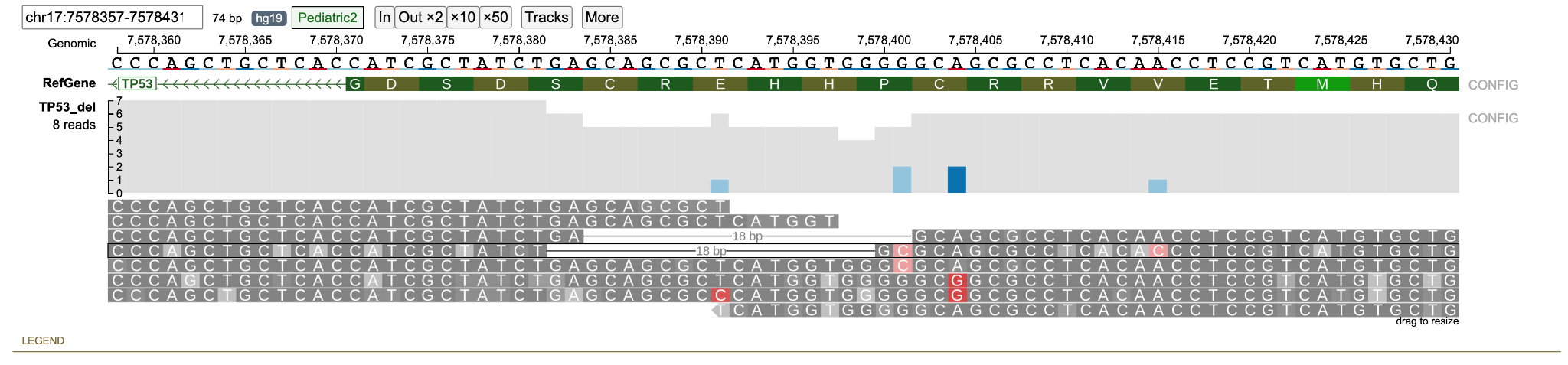


#### Vertical zoom: examining subset of reads

ppBAM can display up to 7000 reads, and will downsample if the number of reads in a region is over 7000. This is especially helpful for displaying high-depth sequencing data. However, displaying nucleotides from each read for such a large number of reads is not feasible. Therefore, the pixel width of each read is reduced to accommodate all reads in the region (Panoramic view, figure below). When the user clicks on a read, that part of the alignment stack is enlarged to show the nucleotides within each read (Nucleotide view, figure below) stacked near the cursor click. Reads at the top and bottom of the stack can be viewed by scrolling up/down with the scroll-bar. The top/bottom of the green scroll-bar can be adjusted to display more reads on the screen by reducing the individual width of each read. On clicking the gray area of the scroll bar region, the panoramic view is displayed again.


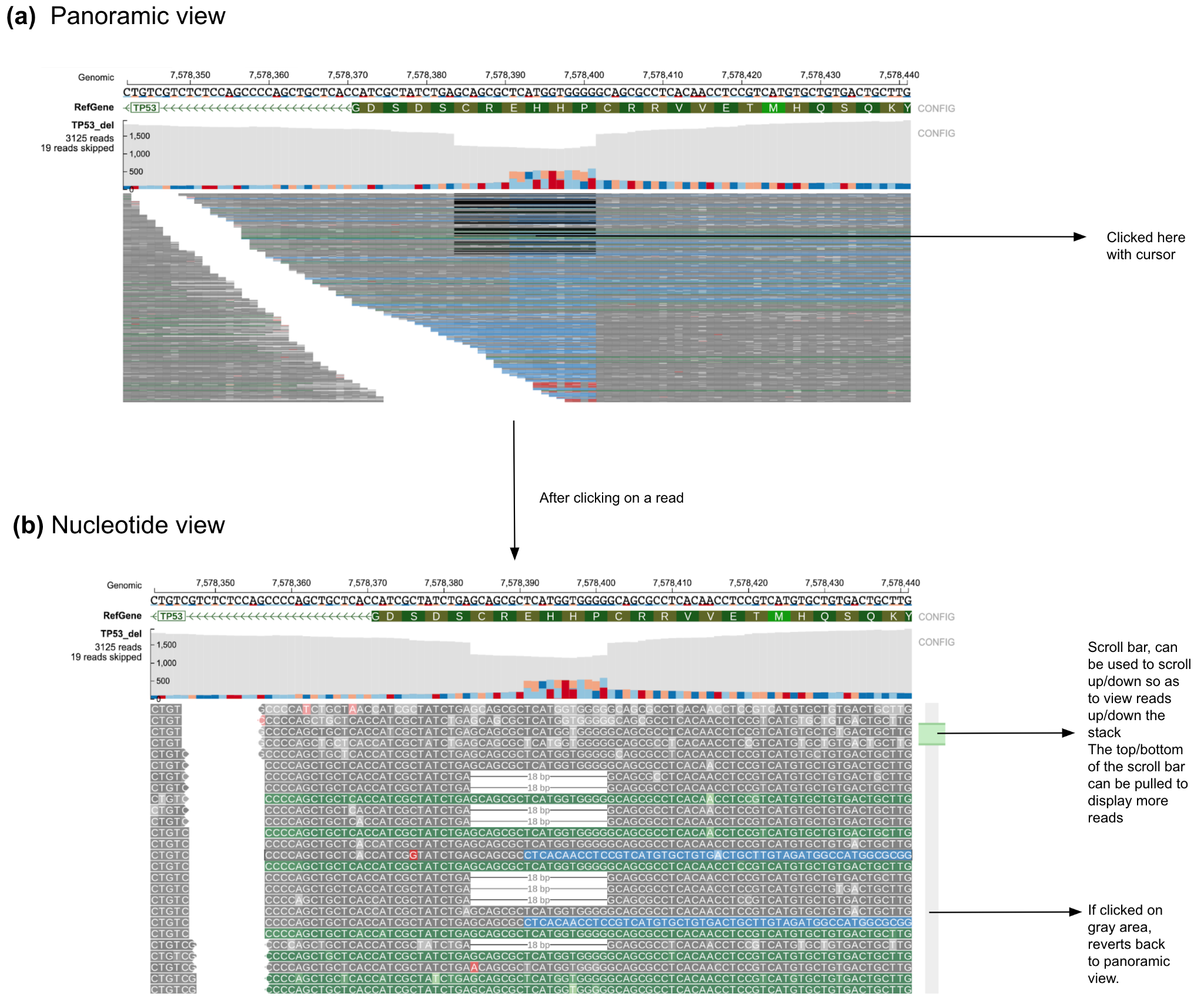


# BAM track configuration panel

The BAM track configuration panel can be accessed by clicking the “CONFIG” option next to the pileup plot. The BAM track configuration panel (shown below) provides buttons for toggling between single-end and paired-end mode. It also provides a check box to show/hide PCR and optical duplicated reads.

## BAM track configuration panel figure


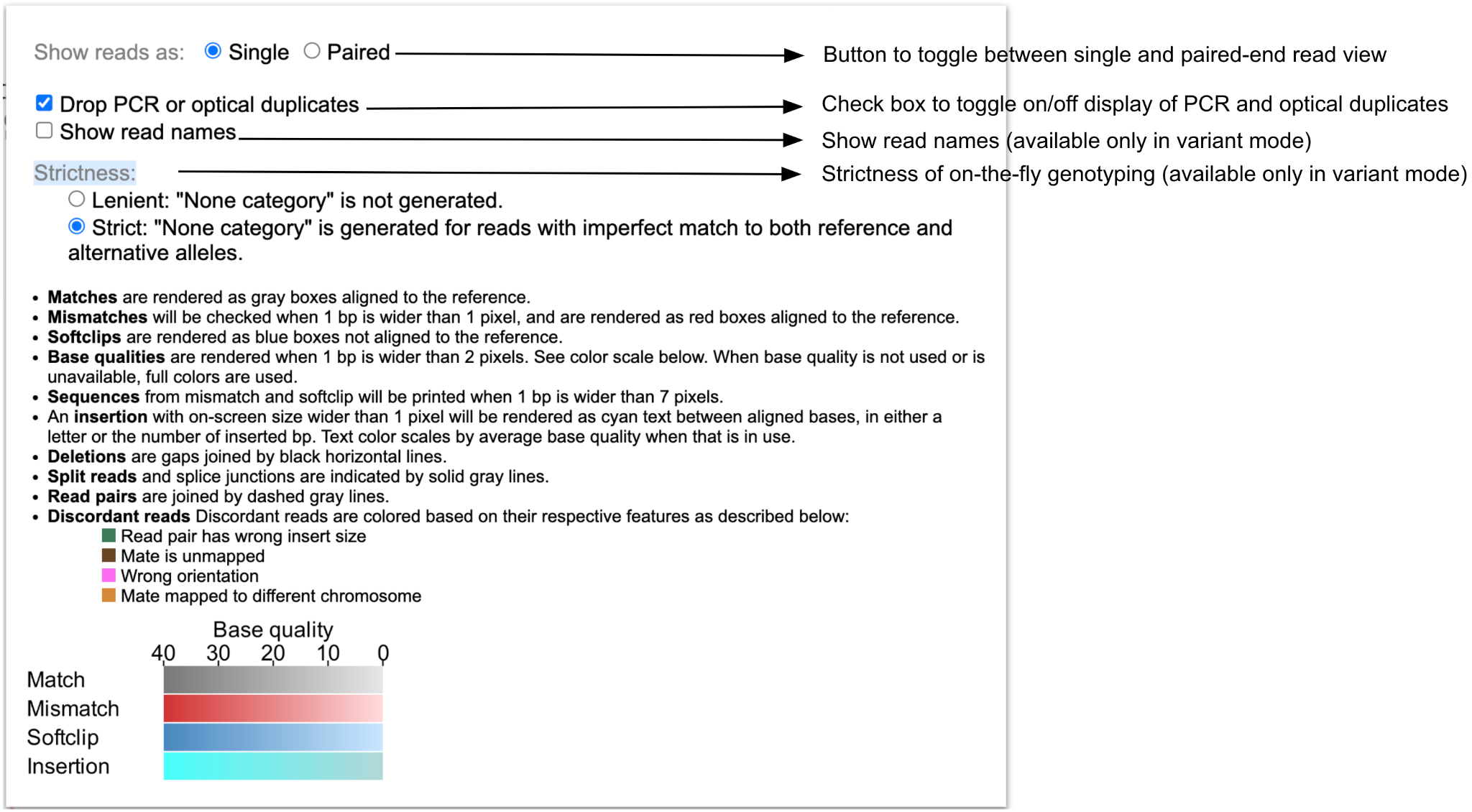


## Single and paired-end read

The [configuration panel](#_pk4jzgmhupjq) (above) provides a toggle to change view between single-end (default) and paired-end view (shown below). [Link](https://proteinpaint.stjude.org/?genome=hg19&block=1&bamfile=crebbp_del,proteinpaint_demo/hg19/bam/crebbp.bam&position=chr16:3800245-3803429) to example shown below. In single-end display each read is displayed individually without displaying any connections with its respective mate. In case of the paired-end display the two paired reads are joined by a gray dotted-line if the coordinates of the two reads do not overlap. When the coordinates of the two read-pairs overlap, the overlapped region is highlighted by a blue line.

Following shows reads in single-end mode.


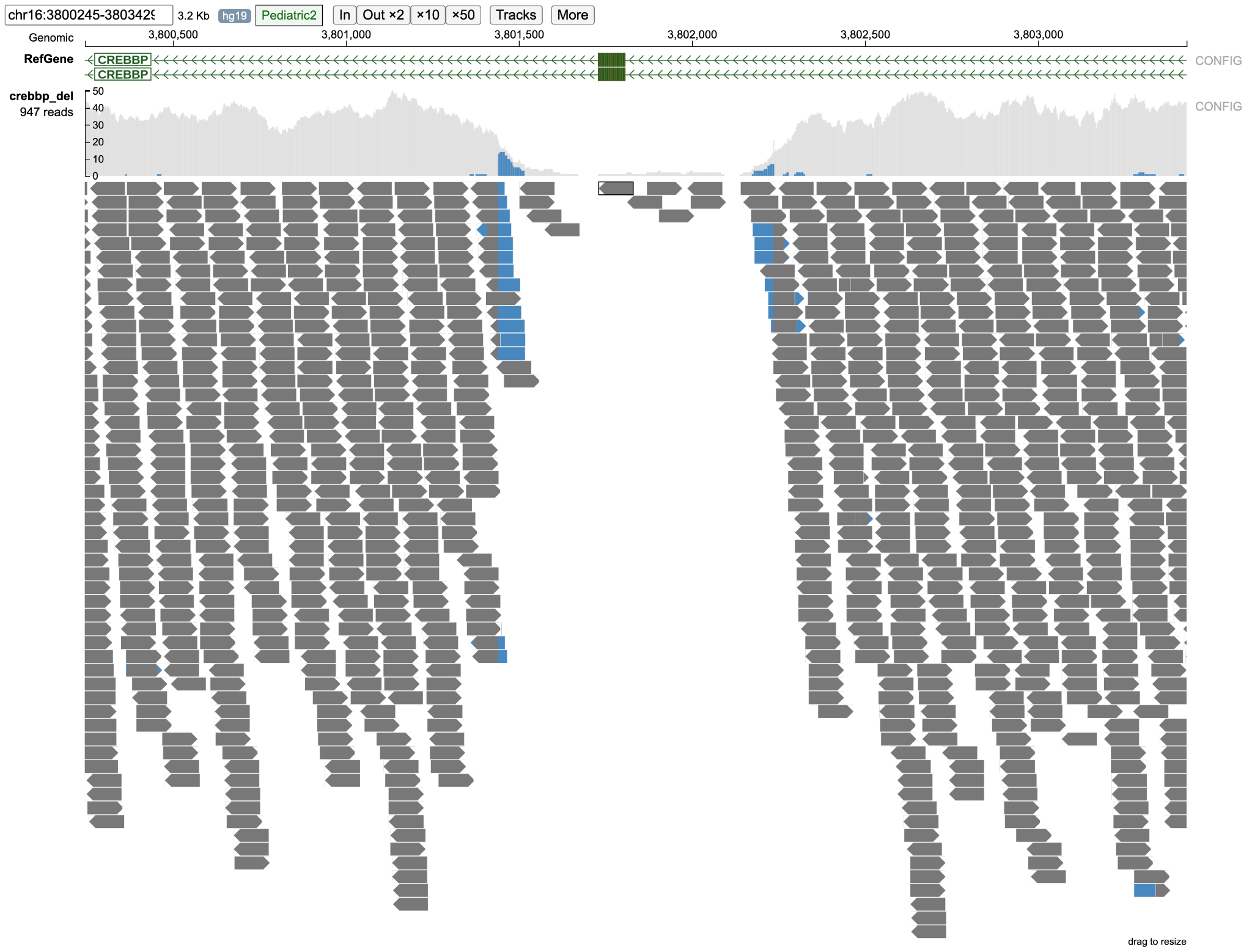


The same track above shows in paired mode.


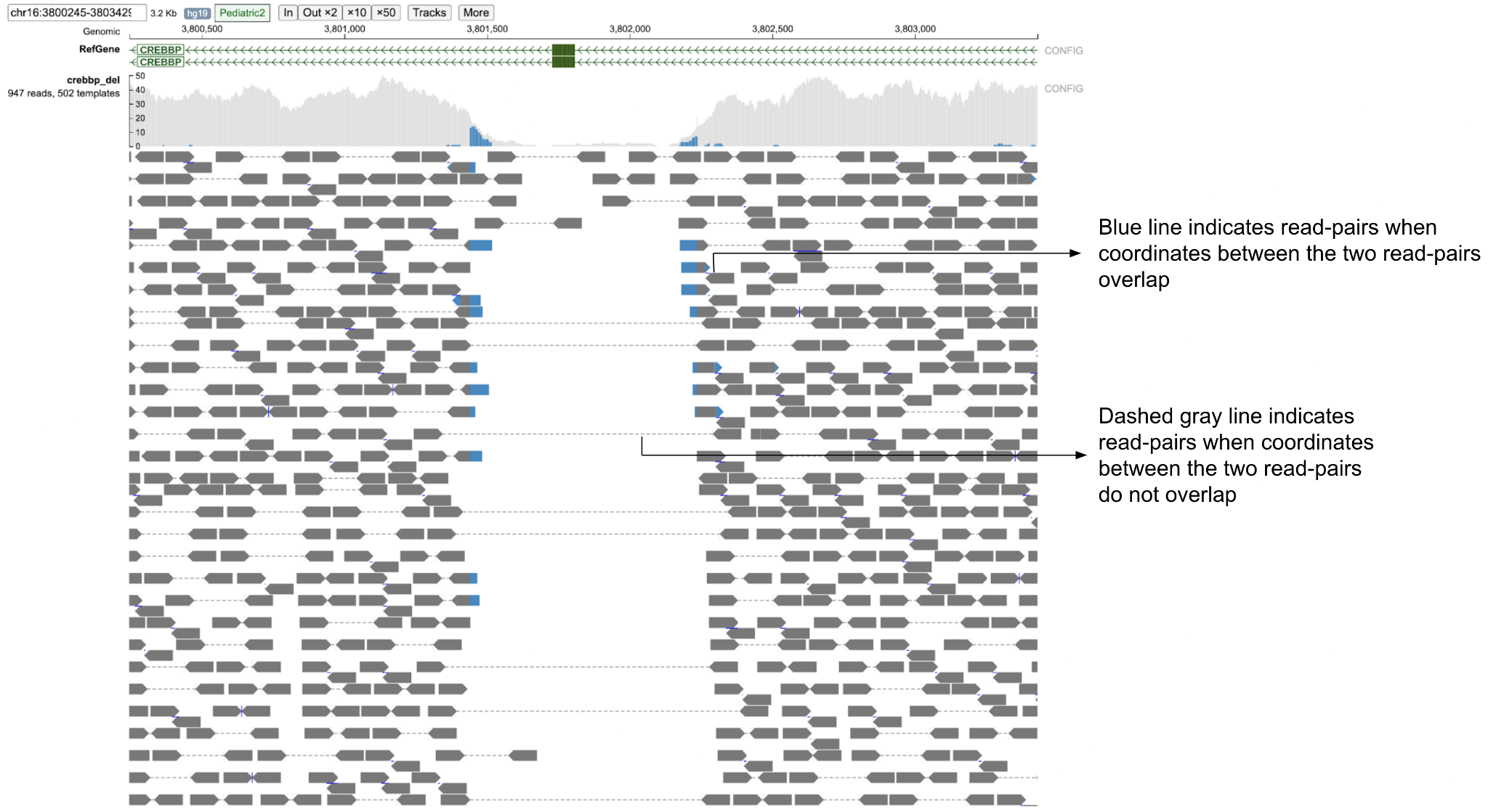


## Show/hide read names

Available only when the [variant](#_8xbzrcxbtx60) field is specified. Checkbox that displays read names on the left side of the [main BAM track as shown below](https://proteinpaint.stjude.org/?genome=hg19&block=1&bamfile=show_read_names,proteinpaint_demo/hg19/bam/show_read_names.bam&position=chr17:7578191-7578591&variant=chr17.7578383.AGCAGCGCTCATGGTGGGG.A&bedjfilterbyname=NM_000546). The read names are only displayed when the main BAM track has [base-pair level resolution](#_x7c2znea1qxu) and nucleotide view ([vertical zoom](#_qxzh89gdrfte) in case of high-depth sequencing data).


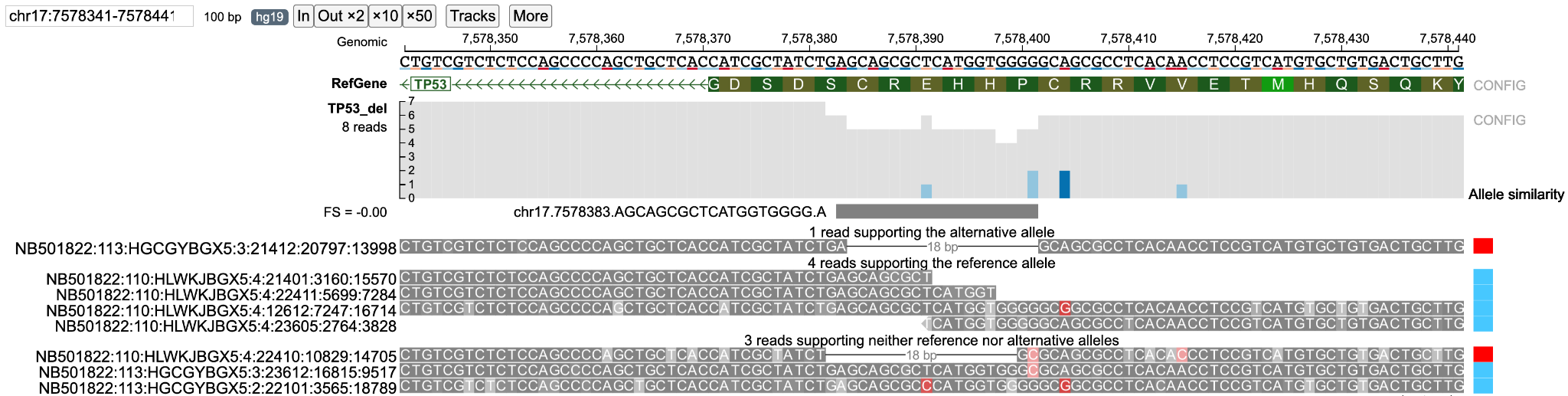


## Displaying PCR and optical duplicated reads


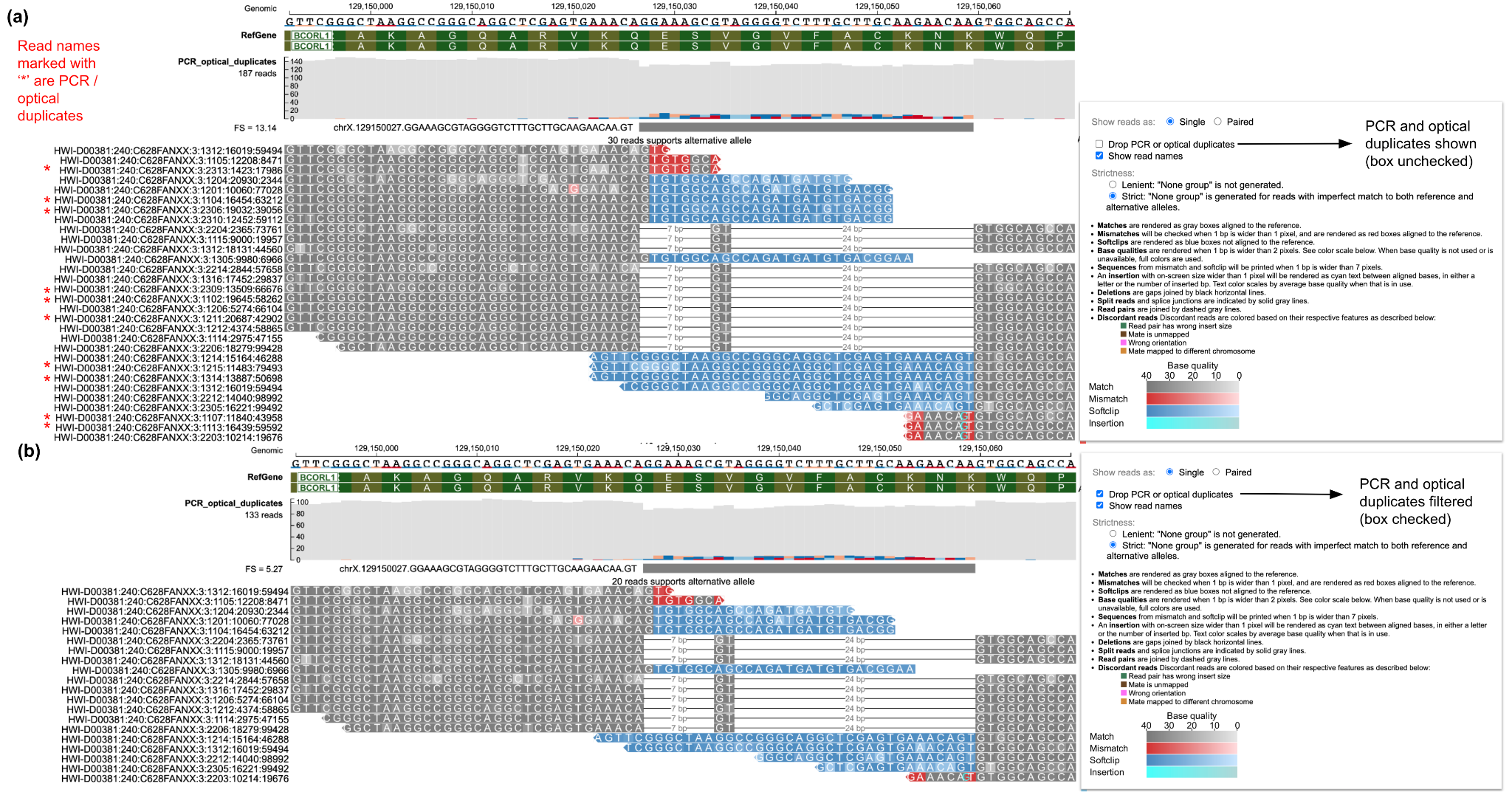


The [check box](#_pk4jzgmhupjq) in the configuration panel can be toggled to switch on/off display of PCR and optical duplicates. [In the above figure](https://proteinpaint.stjude.org/?genome=hg19&block=1&bamfile=long_del,proteinpaint_demo/hg19/bam/PCRopticalduplicates.bam&position=chrX:129150008-129150048&variant=chrX.129150027.GGAAAGCGTAGGGGTCTTTGCTTGCAAGAACAA.GT&Bedjfilterbyname=NM_001184772), a total of 30 reads are shown when PCR/optical duplicates are displayed (Figure a) whereas a total of 20 reads are displayed supporting the alternative allele when PCR/optical duplicates are not displayed (default, Figure b).

## Strictness

[Strictness](#_h2q7c289qfvv) of the on-the-fly genotyping analysis. This option is available when the BAM track is performing on-the-fly genotyping against a variant. The user can toggle between Lenient and Strict (default) mode as shown in the ppBAM [configuration panel figure](#_8dsa4719ln5f).

# Read information panel

For displaying the various features of individual reads, on clicking a particular read (in nucleotide view) opens a new panel displaying the information about the selected read (as shown below).


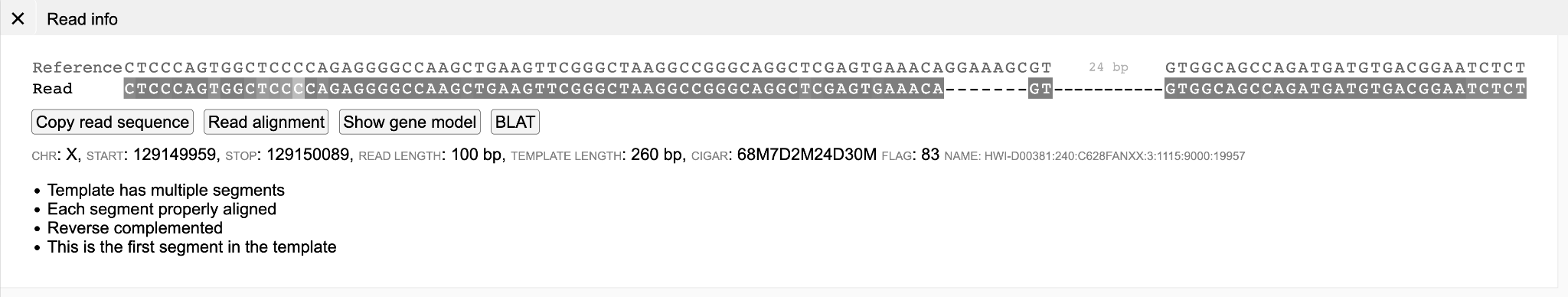


In this panel (as shown above), the top row shows the reference sequence that is aligned to the read. The second row shows the nucleotide sequence of the read. The colors of the nucleotides of the read are based on the CIGAR sequence of the read and follow the color codes as described in the section [color coding of reads](#_m2mkufdeqgt4). In the third row, three clickable buttons are available which have the following functions as described below. The fourth row contains the start, stop, read length, template length, CIGAR sequence, flag and name of read.

## Copy read sequence

Copies the nucleotide sequence of the read being displayed to the computer clipboard so that it can be pasted outside of ppBAM.

## Show gene models

On clicking this button, the gene model (as shown below) (as described for the [ProteinPaint Genome Browser](#_n93ehy8g604w) figure) is displayed.


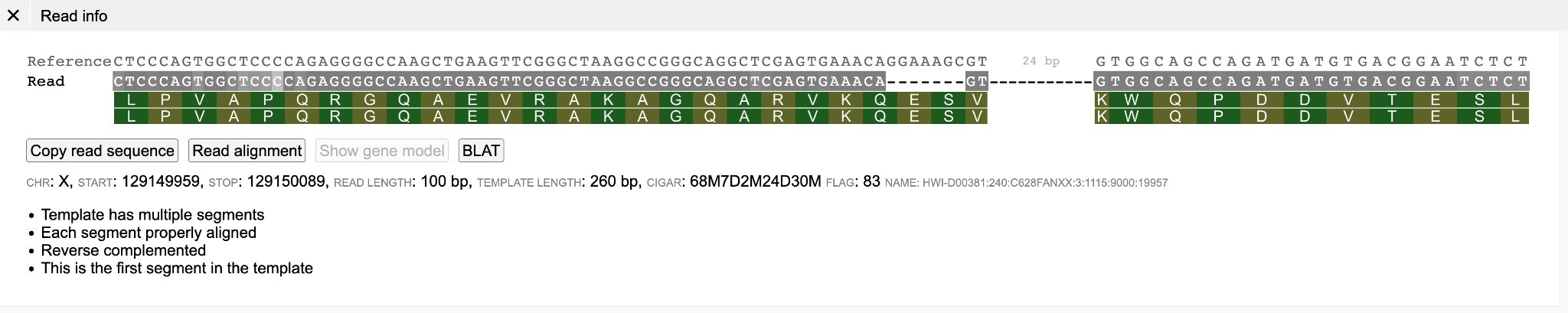


## BLAT

On clicking this button, the read sequence is aligned against the given reference genome build using BLAT (as shown below).


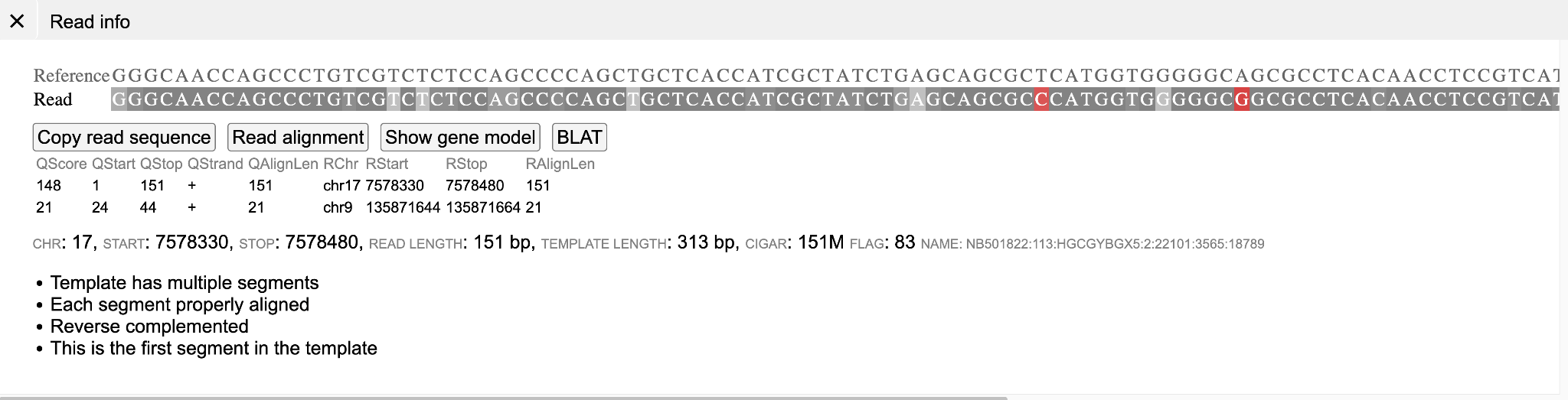


Each of the columns obtained from BLAT alignment are explained below:

### QScore

Score of the BLAT alignment. Generally higher scores mean better alignment

### QStart

Start position of alignment with respect to read i.e. from which nucleotide position the alignment started in the read.

### QStop

Stop position of alignment with respect to read i.e. from which nucleotide position the alignment stopped in the read.

### QAlignLen

Number of nucleotides in the query sequence aligned to the reference genome.

### RChr

Chromosome of reference region aligned.

### RStart

Start position of alignment in reference genome.

### RStop

Stop position of alignment in reference genome.

### RAlignLen

Alignment length in the reference genome.

## Read details

The fourth row contains details about the read present in the BAM file

### CHR

Chromosome ID of the read.

### START

Contains the start position of the read.

### STOP

Contains the stop position of the read.

### READ LENGTH

Contains length of the read.

### TEMPLATE LENGTH

Contains length of the template of which the current read is part of.

### CIGAR

Contains CIGAR sequence of the read.

### FLAG

Contains the flag number (from BAM file) of the read.

### NAME

Contains the name of the read.

## Color coding of reads

1. Paired-end view of deletion in CREBBP gene

**
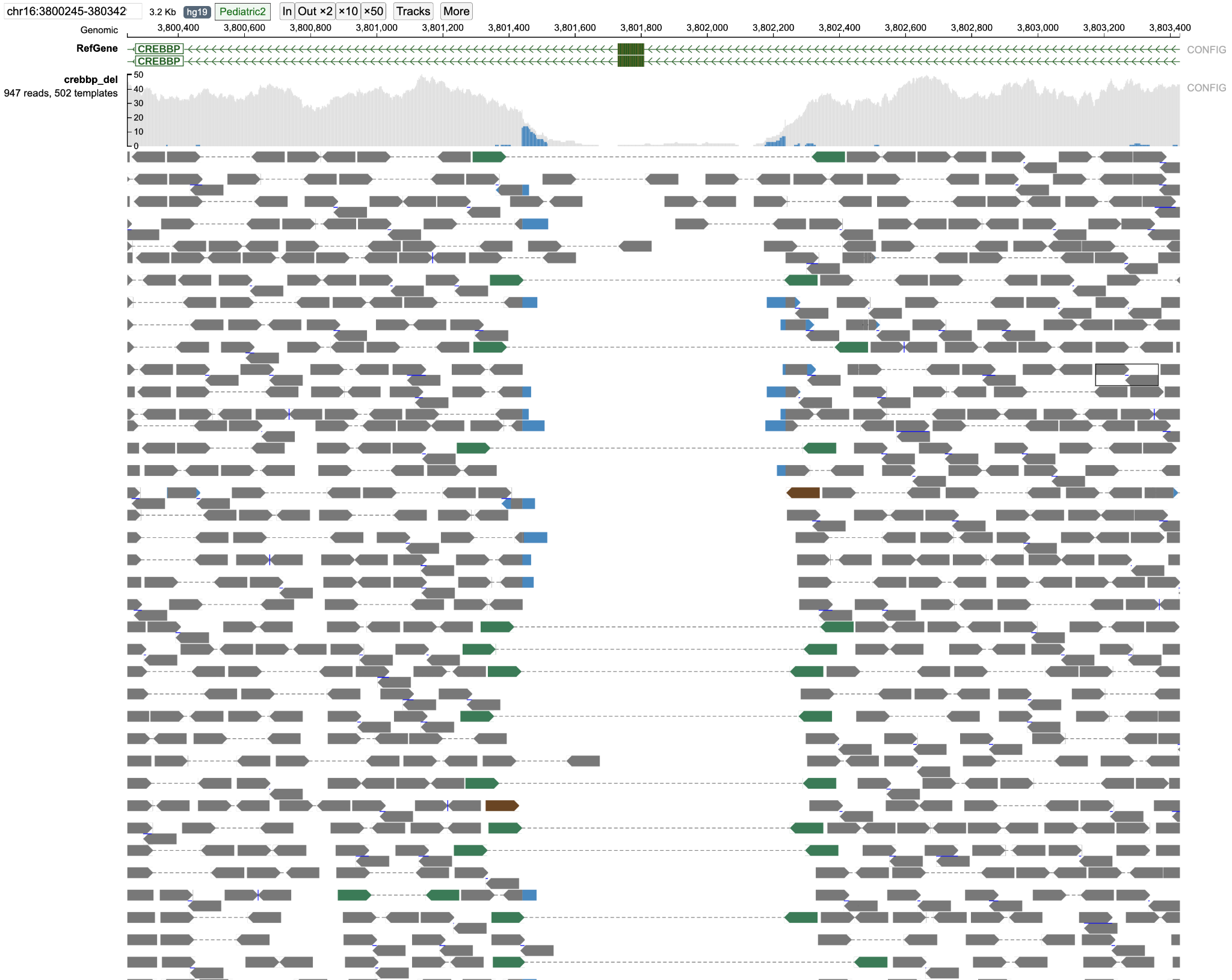
**

1. Base-pair resolution mode showing nucleotides of each of the reads

**
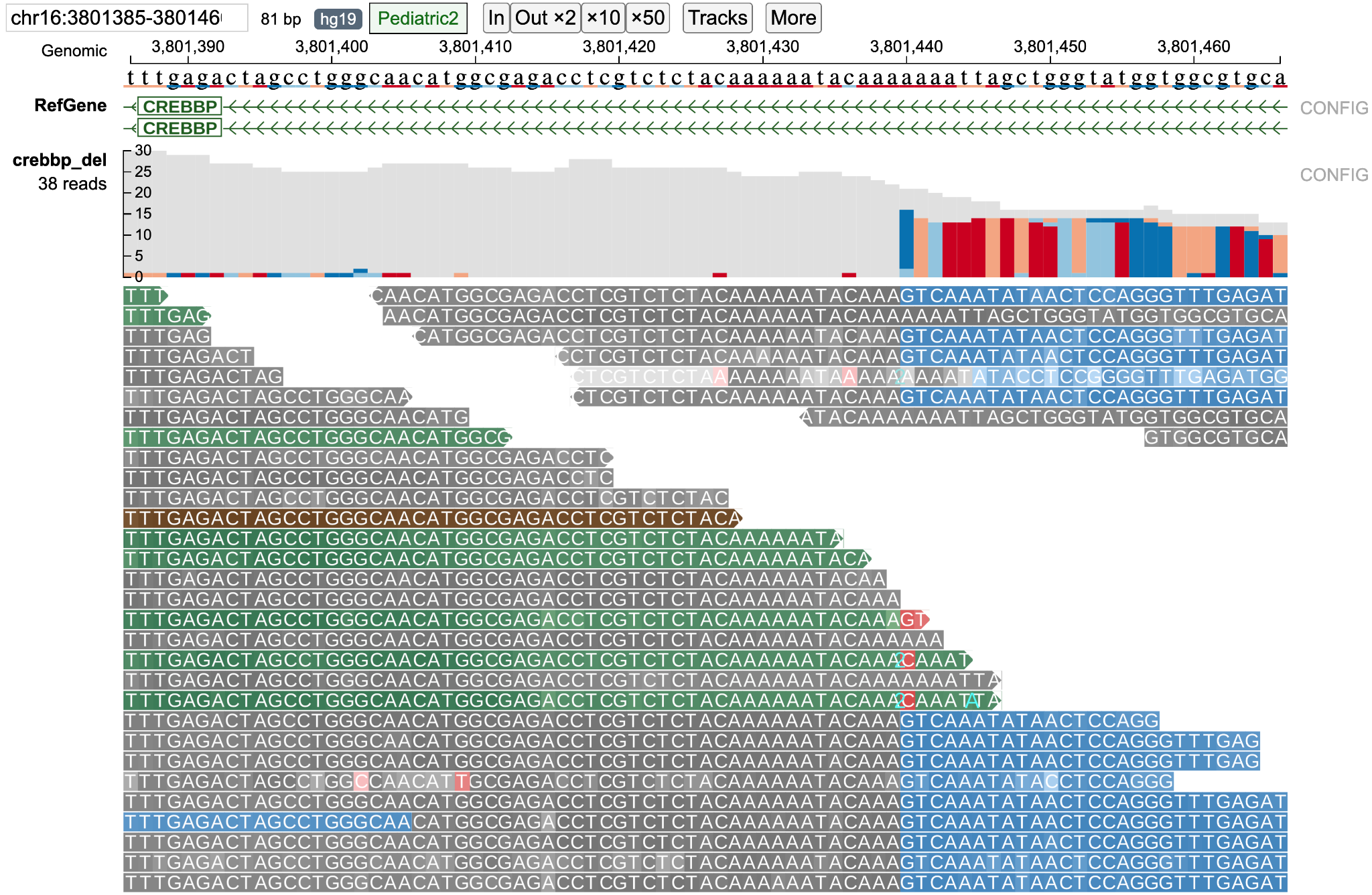
**

In the [figure](https://proteinpaint.stjude.org/?genome=hg19&block=1&bamfile=crebbp_del,proteinpaint_demo/hg19/bam/crebbp.bam&position=chr16:3800245-3803429) above, structural variant deletion in the CREBBP gene is shown. Reads near the vicinity of the deletion have various colors (gray, green, brown and blue) based on their features as explained below. In the paired-end view (a) an overview of the deletion is shown. In Base-pair resolution mode (b) showing nucleotides of each of the reads we can see [softclipped](#_bkq91auo81mr) reads starting [near position chr16: 3,801,439](https://proteinpaint.stjude.org/?genome=hg19&block=1&bamfile=crebbp_del,proteinpaint_demo/hg19/bam/crebbp.bam&position=chr16:3801385-3801466).

Color codes in the background (as shown above) of the read describe the quality of the alignment of the read and its mate (in case of paired-end sequencing). These colors are assigned both on the basis of the CIGAR sequence (if it contains a softclip) and the flag value of both the read and its mate.

### Gray

Presence of gray background nucleotides in a read suggests that both the read (at least part of it) and its mate are properly aligned and the insert size is within expected range (as shown below).


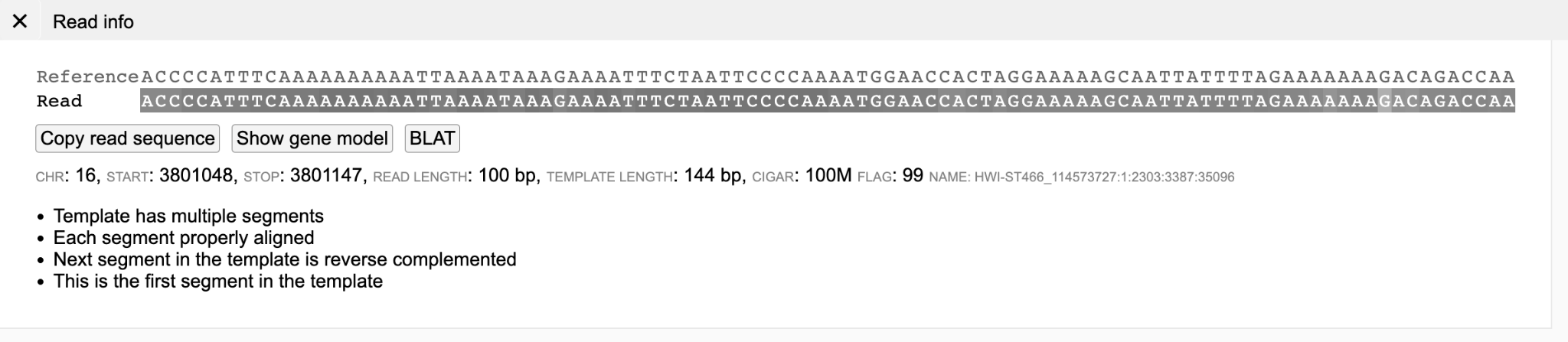


### Blue

Presence of blue-background nucleotides in a read indicates that part of the read is soft clipped (as shown below). The last 42 nucleotides in the read below are softclipped based on CIGAR sequence (58M42S).


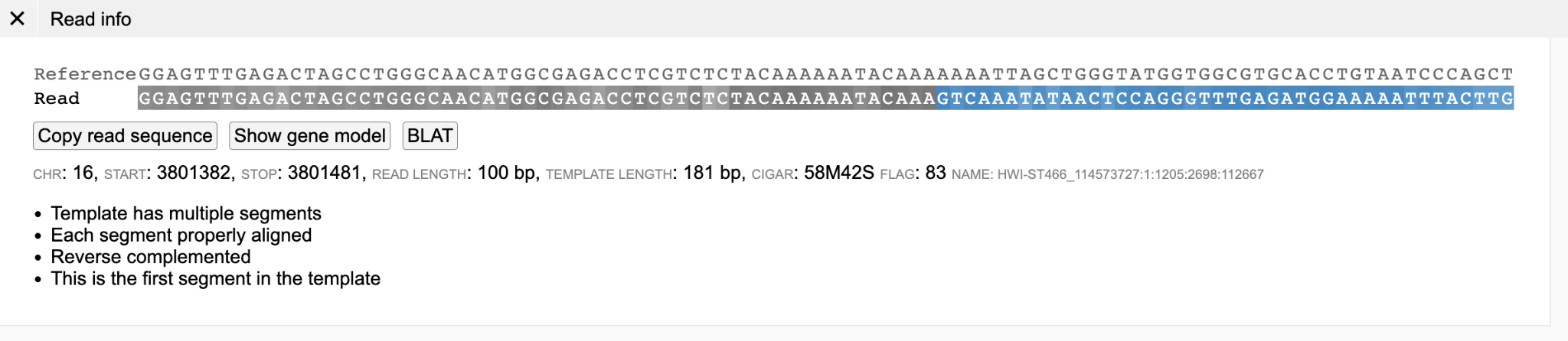


### Brown

A brown colored background (in the main [read alignment plot](#_38dzjdbvti15)) indicates that the mate of the read is unmapped. Such reads have a [flag](#_ggyi4p63lfbk) value that contains the 0x8 bit. In the read information panel on clicking a read with unmapped mate, the current read sequence is displayed along with a button “Show unmapped mate”.


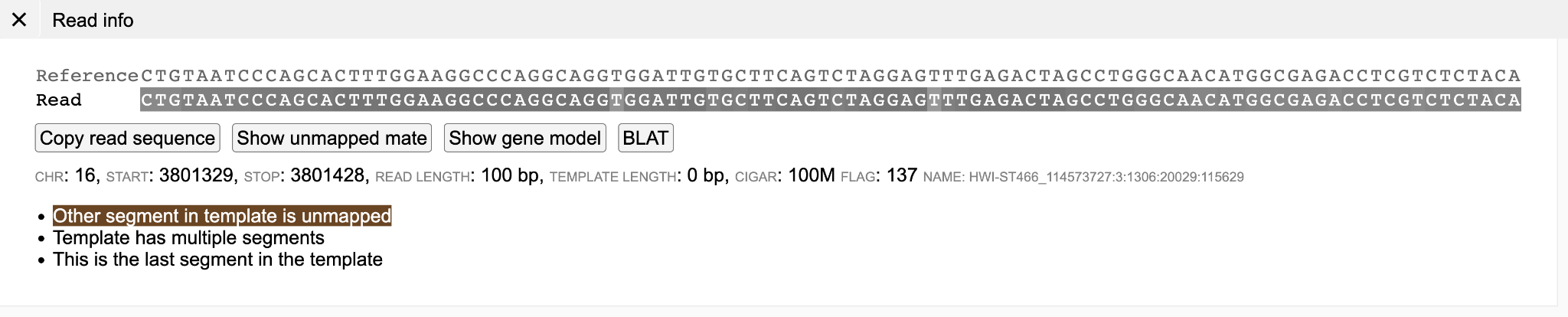


On clicking the button “Show unmapped mate”, the sequence of the unmapped mate is also displayed.


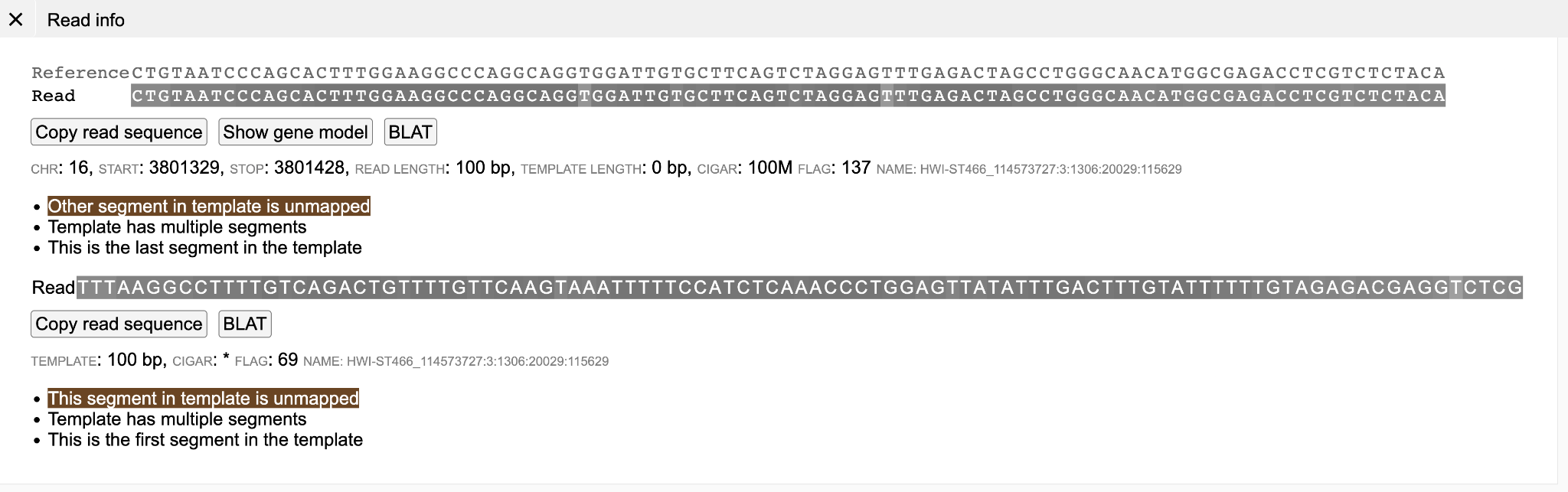


### Green

A green background (shown below) indicates that the template has the wrong insert size. As shown in Figure, the reads labeled green have higher insert size than normal (gray) reads because of the structural deletion. In [paired-end view](#_m2mkufdeqgt4), generally such read-pairs have a much longer gray-dashed line than properly aligned (Gray) read-pairs.


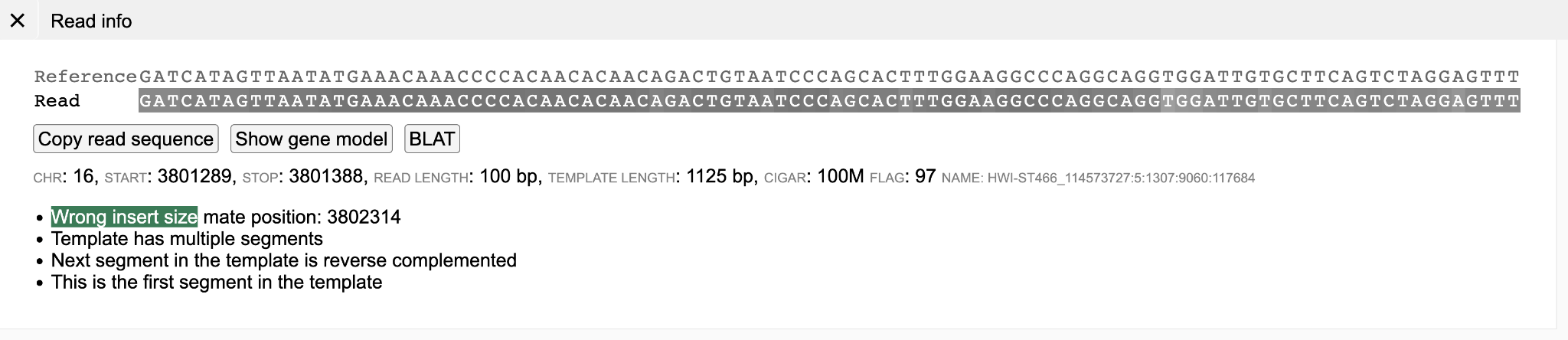


### Pink

A pink color background indicates that the orientation of the read and its mate is not correct. Several orientations are taken into consideration. Here is a [link](https://proteinpaint.stjude.org/?genome=hg19&block=1&bamfile=test,proteinpaint_demo/hg19/bam/CBFB-MYH11.bam&position=chr16:67115893-67117379) to an example of an incorrect orientation. The figure below displays an example of an inversion caused due to CBFB-MYH11 gene fusion found in [Acute Myeloid Leukemia (AML) patients](https://pubmed.ncbi.nlm.nih.gov/32015759/). Here the read and its mate are oriented in the reverse direction (R1R2).

- F1F2 - When both read and its mate are pointing in the forward direction (**-> ->**).
- R1R2 - When both read and its mate are pointing in the reverse direction (**<- <-**).
- F1R2 - When both read and its mate are pointing in forward and reverse direction but are pointing in opposite directions (**<- ->**).


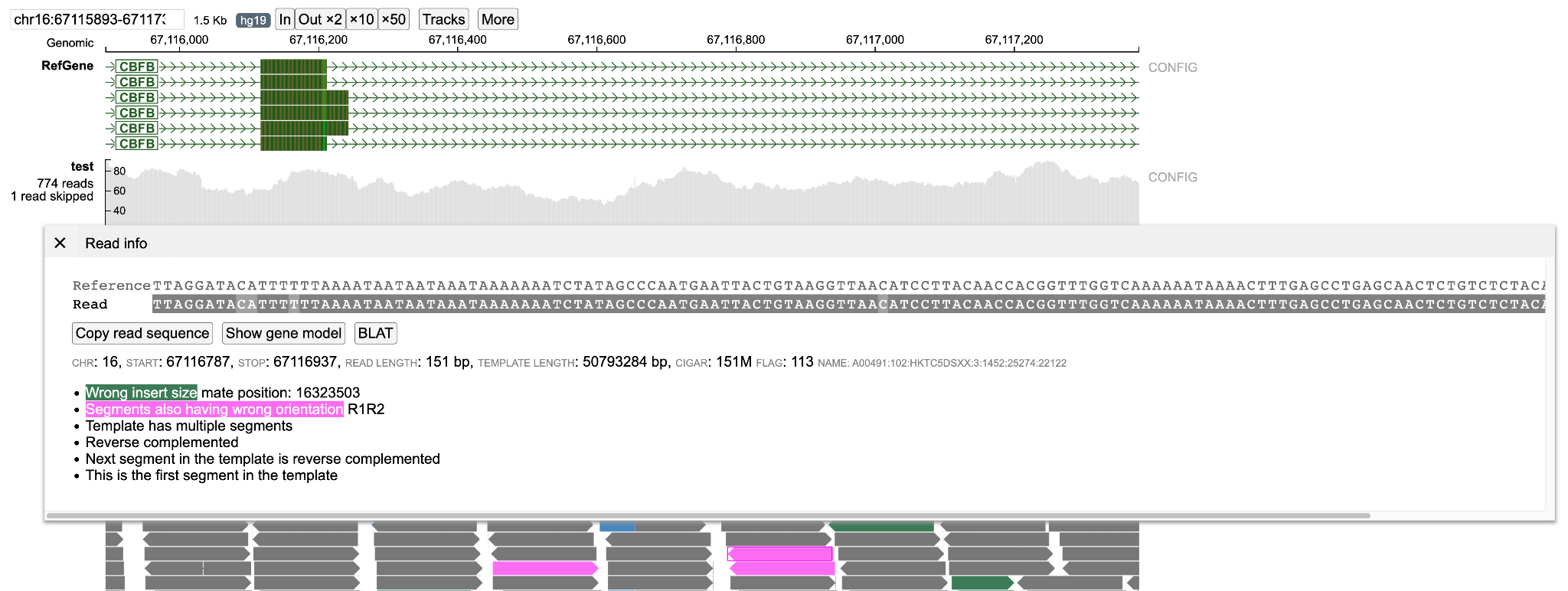


### Orange

Orange background color indicates the read and its mate are mapped in different chromosomes ([as shown below](https://proteinpaint.stjude.org/?genome=hg38&block=1&bamfile=SJACT019118_G1%20WGS,proteinpaint_demo/hg19/bam/discordant_reads.bam&position=chr7:16464-16464&hlregion=chr7:16463-16463)). The displayed read is mapped in chr7:16363-16512 whereas its mate is mapped in chr16.


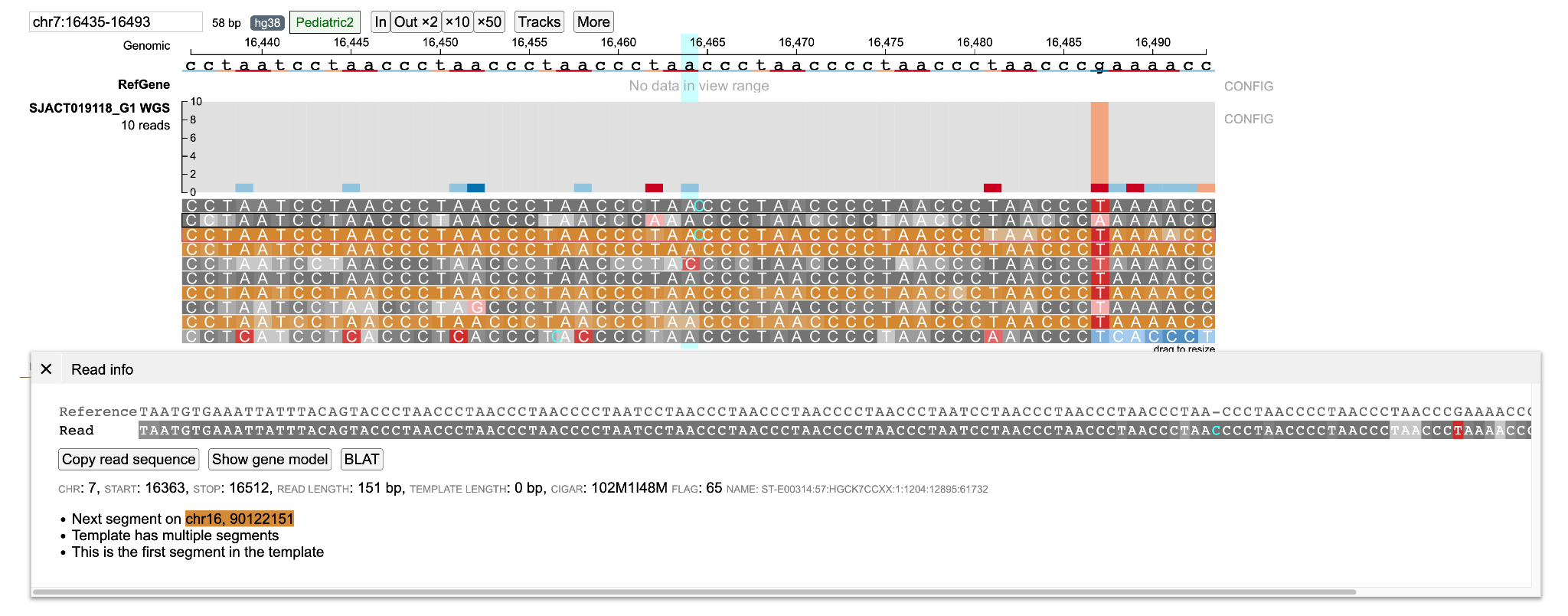


# Variant mode

Variant mode provides an intuitive view of a variant specified by the user inside ppBAM. On specifying the chromosome, position, reference and alternative allele; the reads covering the variant region are displayed and classified into groups supporting the reference allele, alternative allele(s), none (neither reference nor alternative allele) and ambiguous groups. This mode is invoked when the “[variant](#_8xbzrcxbtx60)” field is specified containing the chromosome, position, reference and alternative allele(s) of the variant.

## Read classification into four (or more) groups: Alternative, Reference, None and Ambiguous

For a given variant (SNV or indel), reads mapping to the variant region are classified into Reference, Alternative (possibly multiple alternative alleles when multi-allele variants are queried), None (neither reference nor alternative allele) and Ambiguous (unclassified reads) groups by using the Smith-Waterman alignment (as shown in figure above). Reads are classified into supporting Alternative/Reference allele on the basis of “Allele similarity” which consists of selecting the allele with which the read has the highest identity ratio (number of matched nucleotides/total alignment length) (highest identity ratio highlighted in bold). Reads which do not completely match with neither reference nor alternative alleles are classified into none group (when [strictness](#_h2q7c289qfvv) level = ‘Strict’) and those that have equal allele similarity to both reference and alternative allele (or multiple alternative alleles in case of multi-allele variants) are classified into the [ambiguous](#_rdmthgg2ezwa) group. The barplot on the right displays the “Allele similarity” for each read. This barplot is especially helpful in analyzing reads classified into the none group by indicating the alternative/reference allele with which it has maximum sequence similarity.


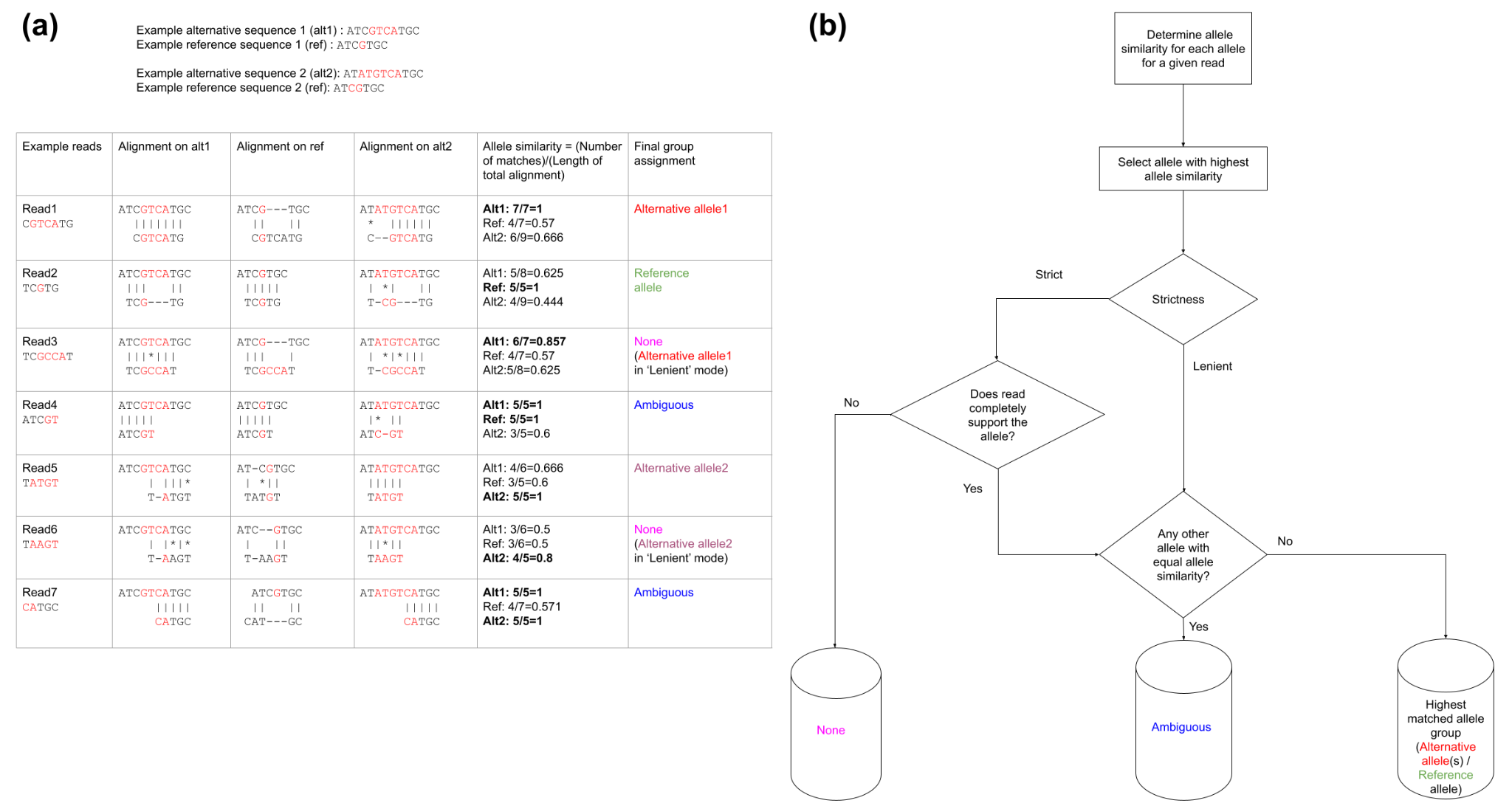


Above figure describes the methodology for classification of reads into Reference, Alternative (possibly multiple alternative alleles), None (neither reference nor alternative allele) and Ambiguous groups. Classification of seven reads are described for a multi-allele variant (G/GTCA) and (CG/ATGTCA). (a) Sequence alignment of various reads to alternative and reference alleles (red colored nucleotides represent alternative and reference allele nucleotides): Read1 and Read2 completely support the alternative allele-1 and reference allele respectively. Read3 has the highest sequence similarity to the alternative allele-1 but has a mismatch and is therefore classified into the none group when [strictness](#_h2q7c289qfvv) = ‘Strict’ and classified into alternative allele-1 when [strictness](#_h2q7c289qfvv) = ‘Lenient’. Read4 has equal similarity to both reference and alternative allele-1 and is classified into the [ambiguous](#_rdmthgg2ezwa) group. Read5 has the highest allele similarity and a complete match to alternative allele-2. Read6 has the highest sequence similarity to the alternative allele-2 but has a mismatch and is therefore classified into the none group when [strictness](#_h2q7c289qfvv) = ‘Strict’ and classified into alternative allele-2 when [strictness](#_h2q7c289qfvv) = ‘Lenient’. Read7 has equal allele similarity to alternative allele-1 and alternative allele-2 and is classified into the [ambiguous](#_rdmthgg2ezwa) group (b) A generalized flow chart for classifying reads into (possibly) multiple alternative/reference alleles using “Allele similarity” values and the “Strictness” setting. The “None” group contains reads which do not support neither reference nor alternative allele(s) will be created only when the “Strictness” setting is set to “Strict” (default). Ambiguous group consists of reads that have equal allele similarity to two (or more) alleles.

## Ambiguous reads

In certain indels such as in the [TP53 example](https://proteinpaint.stjude.org/?genome=hg19&block=1&bamfile=TP53_del,proteinpaint_demo/hg19/bam/TP53_del.bam&position=chr17:7578191-7578591&variant=chr17.7578383.AGCAGCGCTCATGGTGGGG.A&bedjfilterbyname=NM_000546), certain reads in the variant region can have equal similarity towards both the reference and alternative allele. A large number of ambiguous reads are on the left-side of the indel (Fig. a below) because the deletion starts with the sequence GCAGCGC which is also found in the right flanking sequence resulting in equal similarity to both reference and alternative alleles for any read ending within this part of the indel region (as shown in figure below). On viewing the read alignment for the ambiguous read (Fig. b below) through the read information panel, it is observed that the read has equal similarity to both reference and alternative alleles. Nucleotides highlighted in red indicate those which are part of reference/alternative allele. In case of reads in the ambiguous group, the allele similarity plot shows colors corresponding to each allele with which there are equal sequence similarities. In case of single-allele variants, all ambiguous reads have both the color of the reference and alternative allele. In case of [multi-allelic variants](#_rxqat4my59rq), it is possible for a read to have more than two colors when there is ambiguity between multiple alternative alleles.


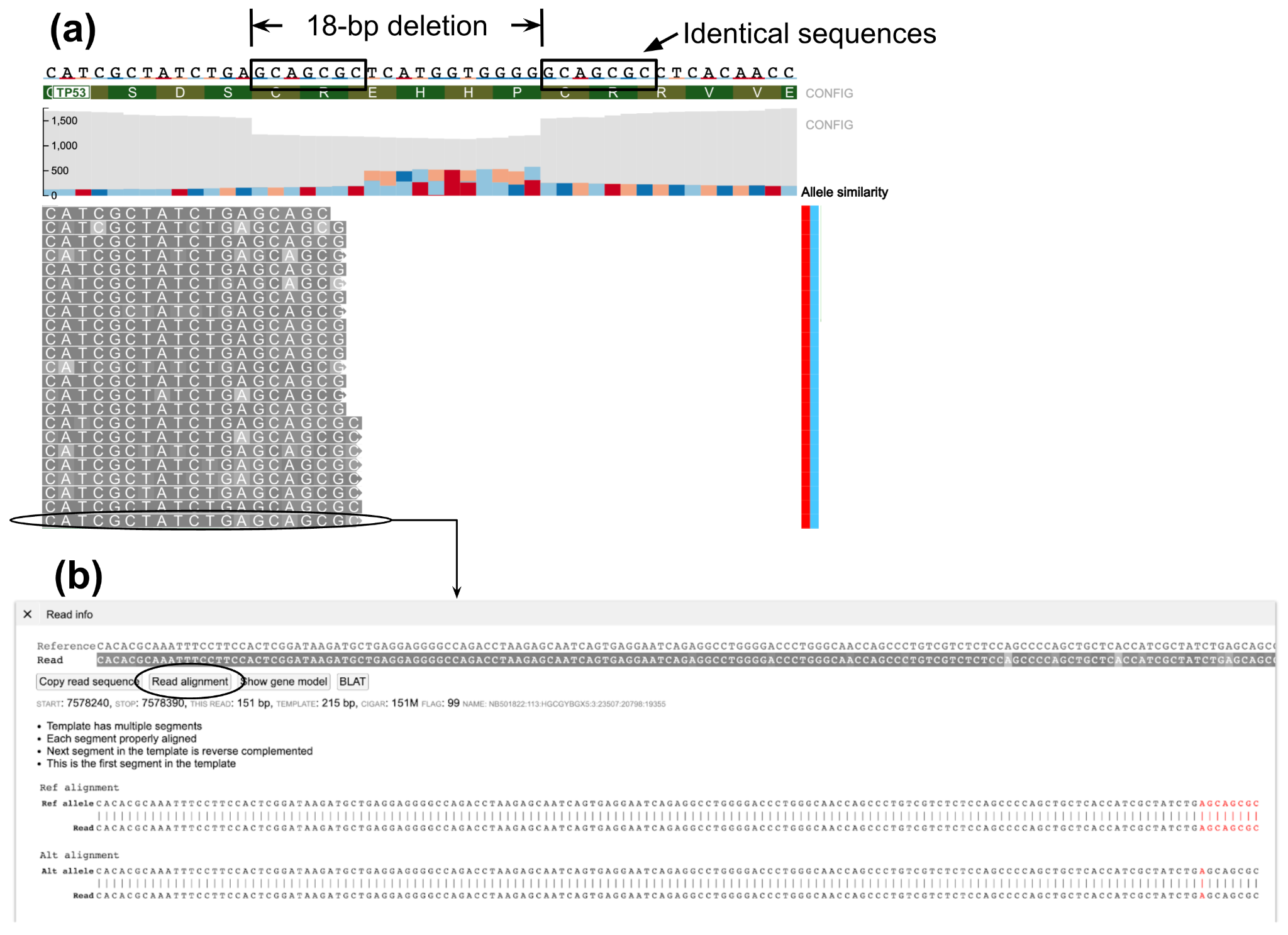


## Fisher-strand analysis to check for strand bias in variants

Fisher-strand (FS) analysis on ratio of forward/reverse strand reads in the alternative and reference groups can help in detecting possible [strand bias](https://gatk.broadinstitute.org/hc/en-us/articles/5358832960539-FisherStrand) that may be present in the variant of interest (shown only for single-allele variants). The FS score is the phred-scaled p-value from the fisher test of the contingency table consisting of forward/reverse strand reads from both the alternative and reference alleles (as shown in figure below). To increase performance for high-depth sequencing examples, when the sequencing depth is greater than 300 the chi-square test is used (if equal or lower than this number, the fisher’s exact test is used). If FS score is [greater than 60](https://gatk.broadinstitute.org/hc/en-us/articles/360035890471), the FS score is highlighted in red (as shown below) indicating that there may be a possible strand bias in the variant.


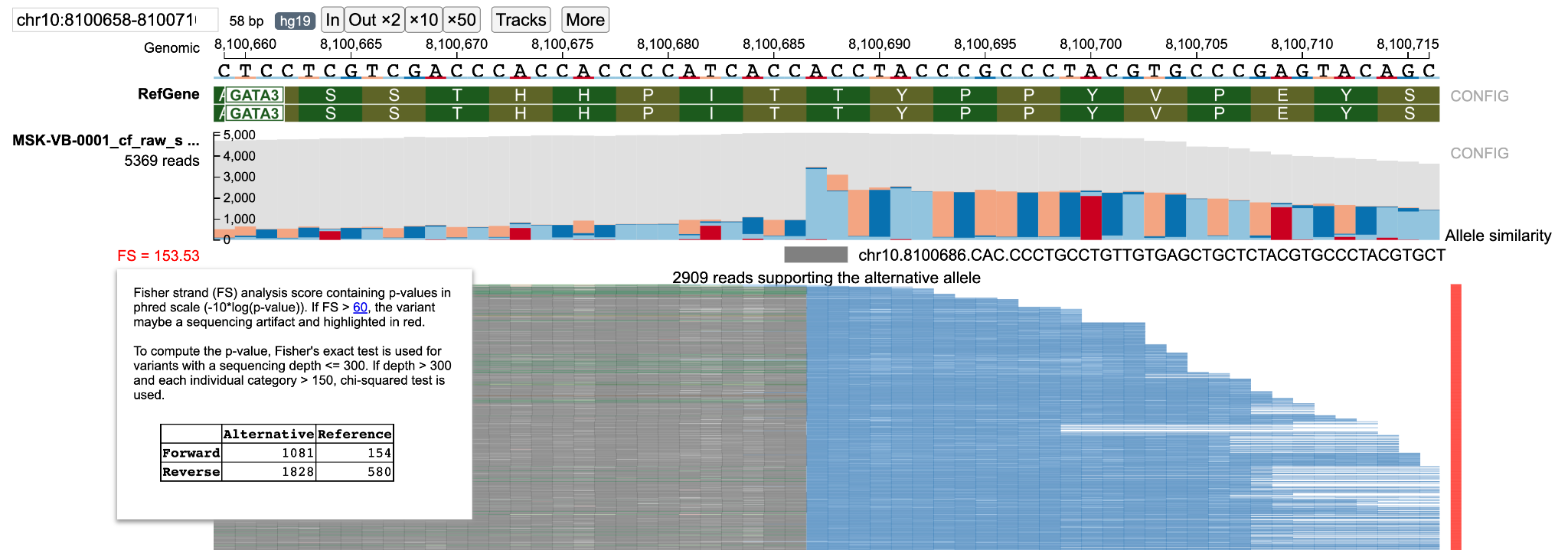


In the figure above, an example of a [complex indel](https://proteinpaint.stjude.org/?genome=hg19&block=1&bamfile=strand_bias,proteinpaint_demo/hg19/bam/strand_bias.bam&position=chr10:8100668-8100707&variant=chr10.8100686.CAC.CCCTGCCTGTTGTGAGCTGCTCTACGTGCCCTACGTGCT) is shown containing fisher strand bias. The FS score is highlighted in red indicating this particular variant may contain strand bias.

## Strictness in on-the-fly genotyping

The user can also optionally change the strictness of the algorithm to Lenient/Strict (default) from the ppBAM [configuration panel](#_pk4jzgmhupjq). For strictness level = ‘Lenient’, reads are classified based on higher sequence similarity to reference/alternative allele. In case of strictness level = ‘Strict’, the exact sequence of the reference/alternative allele in the read is compared against the allele sequence given by the user. Reads that do not match either allele are classified into the none group. In case of the reads in the none group (when strictness level = ‘Strict’), the allele similarity plot shows the color of the allele to which the read has maximum sequence similarity.

The lenient strictness level can be helpful, when the user wants a lenient estimate of the number of reads supporting the particular indel of interest or when the user is confident that only one alternative allele exists. This can also be helpful when there are reads with low base-pair quality calls near the variant region. In contrast when the strictness level is set to ‘Strict’, a more conservative estimate of the read support is provided for each allele and may indicate the presence of a wrong variant call (if present) or may indicate presence of multiple alternative alleles.

In case of the TP53 deletion example, select reads with wrong base pair calls are [shown](https://proteinpaint.stjude.org/?genome=hg19&block=1&bamfile=TP53_del,proteinpaint_demo/hg19/bam/TP53_del.wrongbp.bam&position=chr17:7578371-7578417&variant=chr17.7578383.AGCAGCGCTCATGGTGGGG.A&bedjfilterbyname=NM_000546). For strictness level = ‘Lenient’, there are two reads that support the alternative allele. However, read NB501822:110:HLWKJBGX5:4:22410:10829:14705 has a wrong base pair call at position 7578401. When the strictness level is changed to ‘Strict’, this read is classified into the none group. Similarly, reads NB501822:113:HGCGYBGX5:3:23612:16815:9517 (wrong base-pair call at 7578401) and NB501822:113:HGCGYBGX5:2:22101:3565:18789 (wrong base-pair call at 7578391) are classified in the reference allele group when strictness level = ‘Lenient’ but are classified into the none group when strictness level is set to ‘Strict’.

The ‘Lenient’ strictness level is generally only helpful in cases where only one alternative allele is present as it assumes only the given reference and alternative allele are the only possible cases. For multi-allelic variants or when a region has a large number of reads with low phred base-pair quality nucleotides, the ‘Strict’ (default) level should be used.

## Realignment using Clustal Omega

In the original alignment shown in the main BAM track view, all the reads are aligned against the reference genome. Therefore, in the alternative allele group reads may be mapped differently although they have the same sequence in the variant region. For example, in the reads supporting the alternative allele in the [TP53 example](https://proteinpaint.stjude.org/?genome=hg19&block=1&bamfile=TP53_del,proteinpaint_demo/hg19/bam/TP53_del.bam&position=chr17:7578191-7578591&variant=chr17.7578383.AGCAGCGCTCATGGTGGGG.A&bedjfilterbyname=NM_000546) , the reads either have mismatches, deletions, soft clips or a combination of all three. Figure (a) shows the complete alternative allele group, whereas in Figure (b) selected set of reads from alternative allele group are displayed displaying various kinds of mapping inconsistencies.


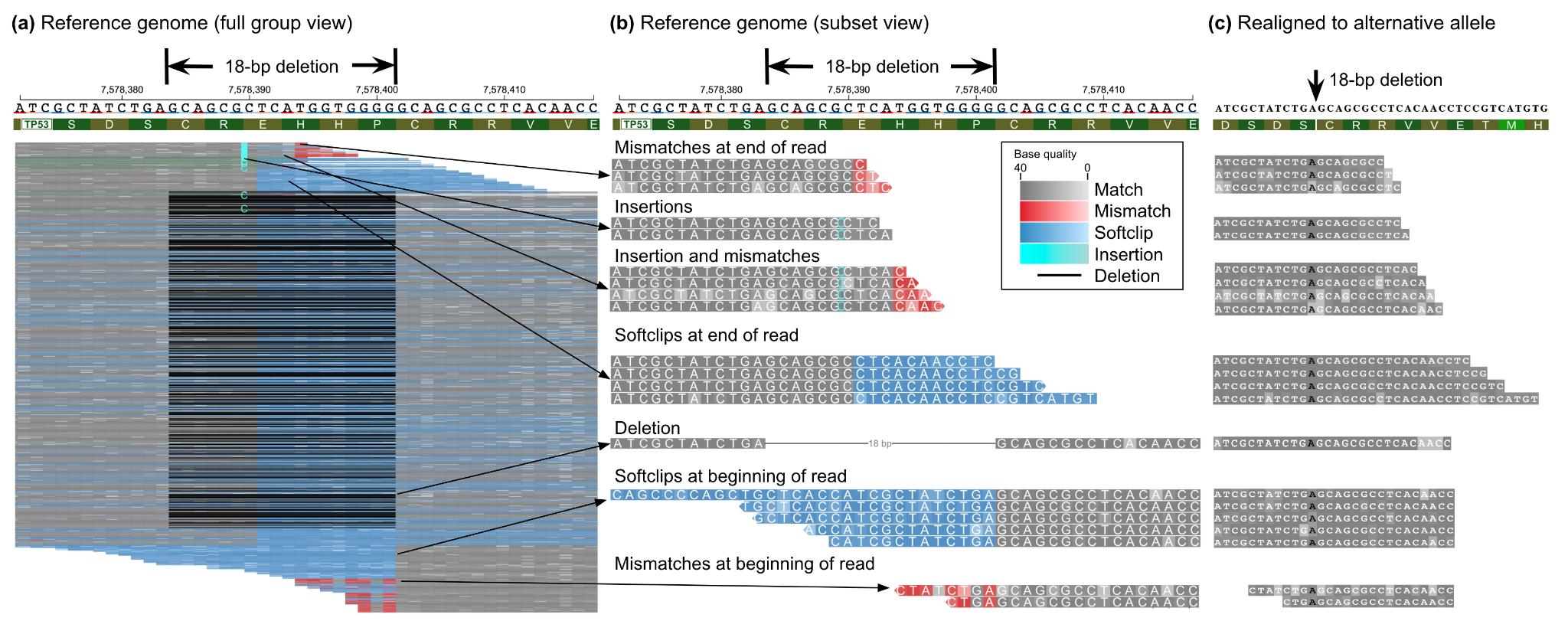


In Figure (c), the reads from (b) are realigned to the alternative allele using Clustal Omega (ClustalO) by clicking on the link showing the number of reads aligned to the alternative allele. This provides an intuitive view confirming the accuracy of the classification of reads to the designated allele. See subset of different reads with same sequence near variant region [mapped differently](https://proteinpaint.stjude.org/?genome=hg19&block=1&bamfile=TP53_del,proteinpaint_demo/hg19/bam/TP53_del.altreads.bam&position=chr17:7578371-7578417&variant=chr17.7578383.AGCAGCGCTCATGGTGGGG.A&bedjfilterbyname=NM_000546).

## Display of read alignment with respect to both reference and alternative allele

In case of reads that are classified into the none group (when strictness level = ‘Strict’) it can be difficult to understand the classification into that group. For example, in case of insertions with the wrong nucleotide (with respect to the predicted alternative allele) the sequence of the inserted nucleotides is not shown in the main BAM track and can only be viewed through the [read information panel](#_7f1mou6qua8k). As an example, a [3bp insertion in CEBPA exon](https://proteinpaint.stjude.org/?genome=hg19&block=1&position=chr19:33792266-33792515&bamfile=CEBPA,proteinpaint_demo/hg19/bam/CEBPA.bam&variant=chr19.33792391.C.CTGC) is discussed below. In Figure (a) (shown below) most reads with 3bp insertion have been classified into the alternative allele. However, there are some reads (as highlighted in Figure a) with 3bp insertions that are classified into the none group. The allele similarity plot suggests that these reads have higher sequence similarity to the alternative allele (and are classified into the alternative group when strictness = ‘Lenient’) and they seem to support the alternative allele. However, when we click on this read (Fig. b) and click on the “Read Alignment” button (which is available only when the [variant](#_8xbzrcxbtx60) field is specified in the URL) the Smith-Waterman alignment of the read with the reference and alternative allele is displayed (Figure b). The indel nucleotides are highlighted in red. In case of the read in the none group (75085182), a mismatch is observed in the indel region between the read and the alternative allele (highlighted by ‘*’ in the alignment row) which explains the classification into the none group. In contrast, the read shown from the alternative allele group (75085106) has a complete match with the alternative allele and is therefore classified into the alternative allele group.

Display of read alignment of the read with respect to both the reference and alternative allele helps provide an intuitive view for describing classification of a read into its respective group.


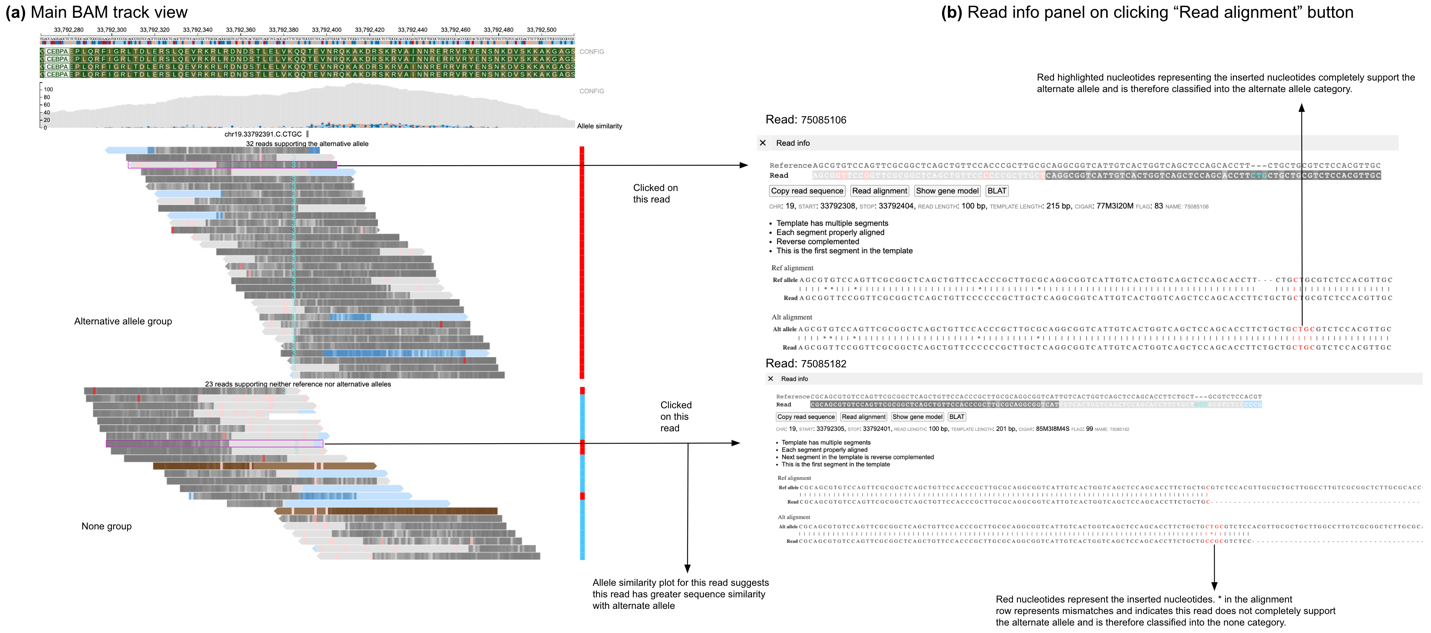


## Classification of multi-allele variants

It is commonly observed that during manual variant review some reads support neither the reference nor the alternative allele. It is possible that some of these reads might represent a different alternative allele. For example in this [variant](https://proteinpaint.stjude.org/?genome=hg19&block=1&position=chr4:55589660-55589870&hlregion=chr4:55589768-55589768&bamfile=Alternate_allele1,proteinpaint_demo/hg19/bam/multi_allele.bam&variant=chr4.55589773.GACAGGC.CTGACAGGCT&bedjfilterbyname=NM_001385285) in the KIT exon, we find that 17 reads have been aligned to the given alternative allele (CTGACAGGCT) (a) but there are also a number of reads in the none group which have similar insertions/mismatches/deletions profile in the variant region (b). For example, all reads marked with “*” symbol have 2T insertion, 1 A/T mismatch and TTACGACA (as shown in (c)) deletion near the variant region whereas reads marked with “-” symbol have ACA/TGT mismatch followed by 3 matches and then T/C mismatch near the variant region. Observing so many reads with a similar alignment profile suggests presence of two additional alternative alleles.


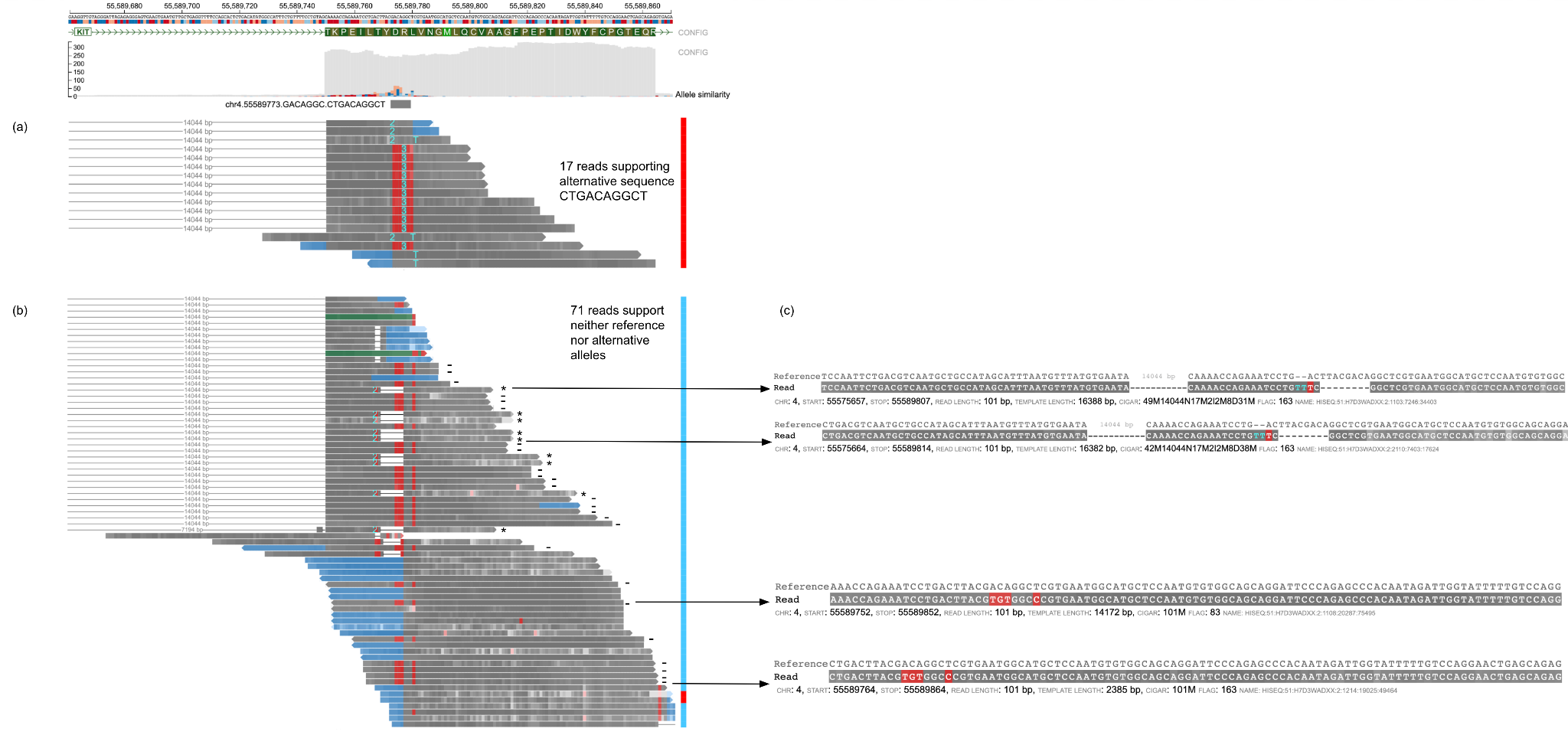


By manually inspecting we find the two new alternative alleles as

chr4.2055589766.GACTTACGACA.GTTTC and chr4.55589774.ACAGGCT.TGTGGCC. On entering these two new alternative alleles in [JSON](#_fzhidfxoli0r) format the multi-allele variant display is invoked. Reads are classified into multiple alternative allele groups in addition to the reference, none and ambiguous groups. Each alternative allele group is represented by a separate color in the allele similarity plot (depicting a multi-allele variant with [three adjacent alleles](http://proteinpaint.stjude.org/?genome=hg19&block=1&position=chr4:55589660-55589870&hlregion=chr4:55589768-55589768&bamfile=multi_allele_variant,proteinpaint_demo/hg19/bam/multi_allele.bam&variant=%7B%22chr%22:%22chr4%22,%20%22variants%22:%5B%7B%22pos%22:55589773,%20%22ref%22:%20%22GACAGGC%22,%20%22alt%22:%20%22CTGACAGGCT%22%7D,%7B%22pos%22:%2055589766,%20%22ref%22:%20%22GACTTACGACA%22,%22alt%22:%22GTTTC%22%7D,%7B%22pos%22:55589774,%22ref%22:%22ACAGGCT%22,%22alt%22:%22TGTGGCC%22%7D%5D%7D&bedjfilterbyname=NM_001385285&strictness=1)) shown in the figure below.


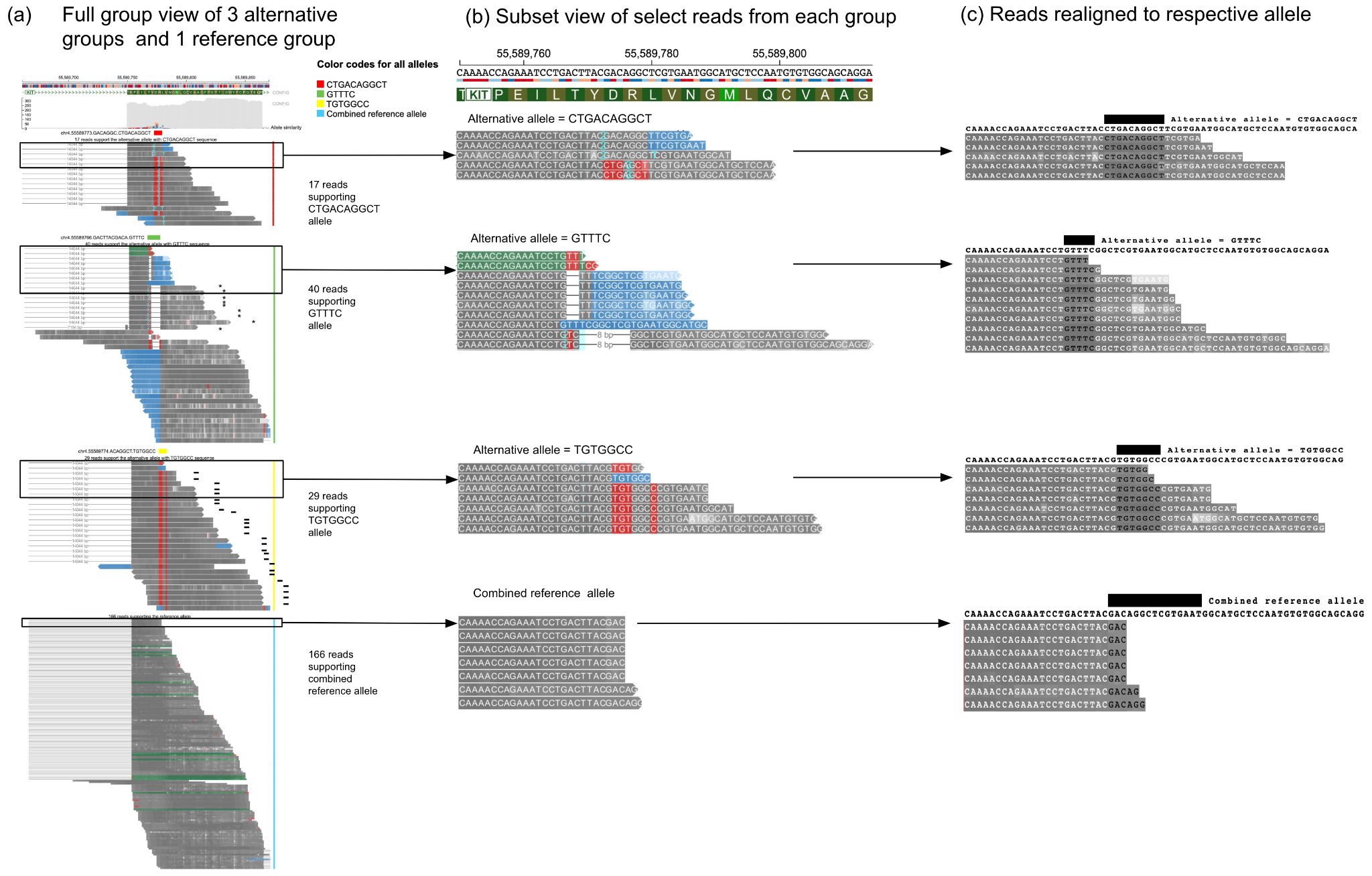


Reads representing each of the three alternative alleles are classified into separate groups in (a). The reads marked with symbol “*” and “-” are now in separate groups confirming the presence of two additional adjacent variants. In (b) select sets of reads from each group have been shown which are aligned differently (in case of alternative allele groups) in the variant site although they represent the same allele. Realignment of those reads to their respective alleles in (c) shows that they all represent the same allele. Since each of three alternative alleles overlap with each other, the reference allele from each variant is combined to highlight all nucleotides (combined reference allele) covered by the reference allele from all the three variants.
